# Effects of myocardial ischemia/reperfusion injury on plasma metabolomic profile during aging

**DOI:** 10.1111/acel.13284

**Published:** 2020-12-29

**Authors:** Claudio de Lucia, Michela Piedepalumbo, Lu Wang, Fausto Carnevale Neto, Daniel Raftery, Erhe Gao, Domenico Praticò, Daniel E. L. Promislow, Walter J. Koch

**Affiliations:** ^1^ Center for Translational Medicine Lewis Katz School of Medicine Temple University Philadelphia Pennsylvania USA; ^2^ Department of Environmental and Occupational Health Sciences University of Washington Seattle Washington USA; ^3^ Department of Anesthesiology and Pain Medicine Northwest Metabolomics Research Center University of Washington Seattle Washington USA; ^4^ Alzheimer's Center at Temple Lewis Katz School of Medicine Temple University Philadelphia Pennsylvania USA; ^5^ Department of Biology University of Washington Seattle Washington USA; ^6^ Department of Lab Medicine and Pathology University of Washington School of Medicine Seattle Washington USA

**Keywords:** aging, heart disease, LC‐MS, metabolism, metabolomics, myocardial ischemia

## Abstract

**Background:**

Heart disease is a frequent cause of hospitalization and mortality for elderly patients. A common feature of both heart disease and aging itself is the involvement of metabolic organ alterations ultimately leading to changes in circulating metabolite levels. However, the specific contribution of aging and ischemic injury to the metabolic dysregulation occurring in older adults with ischemic heart disease is still unknown.

**Aim:**

To evaluate the effects of aging and ischemia/reperfusion (I/R) injury on plasma metabolomic profiling in mice.

**Methods:**

Young and aged mice were subjected to a minimally invasive model of I/R injury or sham operation. Complete evaluation of cardiac function and untargeted plasma metabolomics analysis were performed.

**Results:**

We confirmed that aged mice from the sham group had impaired cardiac function and augmented left ventricular (LV) dimensions compared to young sham‐operated mice. Further, we found that ischemic injury did not drastically reduce LV systolic/diastolic function and dyssynchrony in aged compared to young mice. Using an untargeted metabolomics approach focused on aqueous metabolites, we found that ischemic injury does not affect the plasma metabolomic profile either in young or old mice. Our data also demonstrate that age significantly affects circulating metabolite levels (predominantly amino acids, phospholipids and organic acids) and perturbs several pathways involved in amino acid, glucid and nucleic acid metabolism as well as pyridoxal‐5′‐phosphate salvage pathway in both sham and ischemic mice.

**Conclusions:**

Our approach increases our understanding of age‐associated plasma metabolomic signatures in mice with and without heart disease excluding confounding factors related to metabolic comorbidities.

## Graphical abstract

Studying the intersection between aging and heart disease is a key research area as cardiac dysfunction is a common cause of death in older adults. In our study, we evaluated plasma metabolomic profiling in young and old mice subjected to myocardial ischemia/reperfusion or sham operation. Our results confirmed unique metabolomic features of aging and showed that ischemic injury did not affect plasma metabolomic profile neither in young nor old mice.

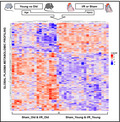

## 1 INTRODUCTION

Heart disease is a frequent and costly condition especially in elderly patients and a common cause of hospitalization and mortality worldwide (Benjamin et al., 2019). Indeed, heart diseases such as coronary artery disease (CAD), myocardial ischemia, and heart failure (HF) show higher incidence with increasing age. In upcoming years, a tremendous increase in the prevalence of HF in older adults is expected as population of those over 65 years of age grows larger. Elderly patients with ischemic HF often display left ventricular (LV) systolic and diastolic dysfunction as well as LV dyssynchrony (de Lucia et al., 2019). Interestingly, the aging process itself also affects cardiac structure, function and molecular signaling pathways, making the heart more vulnerable to stressors that can lead to HF (Chiao and Rabinovitch, 2015). Recent advances in pharmacological treatment and preventive medicine have partially decreased HF incidence and improved outcomes; however, there is still much to be discovered in terms of pathophysiological mechanisms involved in HF development, especially in older adults. This is a complex topic, as several processes in multiple systems have been demonstrated to contribute to cardiac aging development and HF establishment (Houtkooper et al., 2011).

Of note, a common feature of both HF and aging itself is the involvement of metabolic alterations in different organs including liver, muscle, and adipose tissue (Bunning et al., 2020; Houtkooper et al., 2011). Plasma metabolomics is a useful approach to study biological processes occurring in various tissues, which provides large‐scale analysis of the global metabolic state during disease. However, little is known about the specific contribution of aging and myocardial injury to the overall metabolic dysregulation occurring in older adults with ischemic HF. In fact, it is difficult to clarify specific metabolic alterations in the clinical setting since elderly HF patients often exhibit multiple comorbidities (including but not limited to diabetes, metabolic syndrome, and endocrine diseases) (Cirulli et al., 2019; Dharmarajan & Dunlay, 2016).

Hence, we decided to study this phenomenon in the simpler system of an animal model of reperfused myocardial ischemia/infarction. Specifically, we evaluated plasma metabolomic patterns displayed by aged and young mice subjected to ischemia/reperfusion (I/R) injury and age‐matched sham‐operated controls. This approach allowed us to understand age‐associated metabolomic signatures without confounding factors or biases related to metabolic comorbidities.

## 2 RESULTS

### 2.1 Study design, mortality, echocardiographic evaluation, and gravimetric analysis

**Figure 1:**
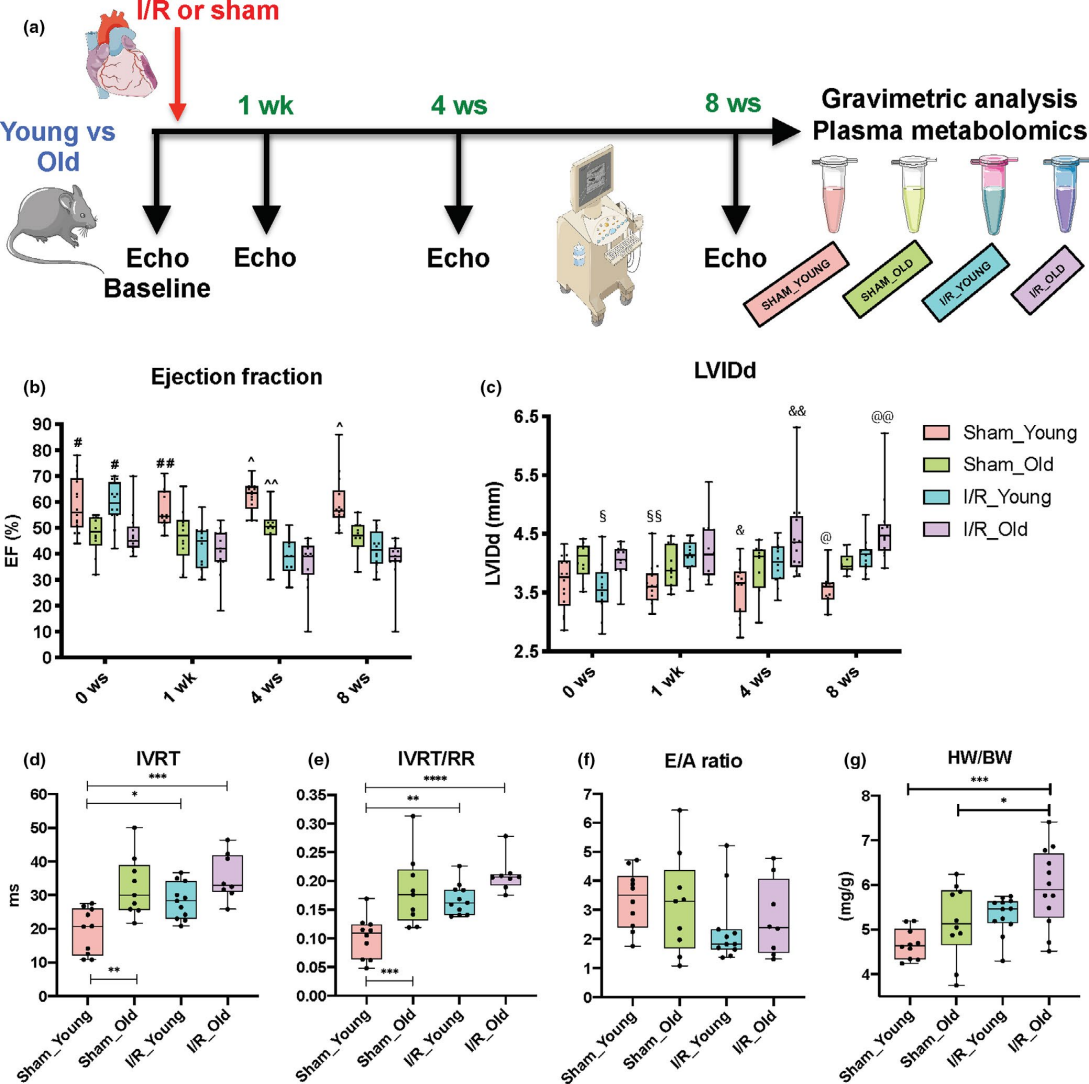
Effects of I/R injury on left ventricular systolic and diastolic function as well as cardiac hypertrophy during aging. Overall design of the 8‐week study in young and old mice subjected to ischemia/reperfusion (I/R) injury or Sham operation (a). Left ventricular function was evaluated with echocardiography (Echo). Left ventricular (LV) ejection fraction (EF) as measured by VEVO Strain Software with modified Simpson method (single plane) (b) and LV internal diameter in diastole (LVIDd) (c) as measured by standard Echo at baseline as well as at 1, 4, and 8 weeks post‐surgery in Sham and I/R groups. Isovolumetric relaxation time (IVRT) (d), IVRT normalized to RR interval (IVRT/RR) (e), E/A ratio (f) (measured with standard Echo) and heart weight to body weight ratio (HW/BW) (g) were evaluated at 8 weeks post‐surgery in Sham and I/R groups. *n* = 8–14 per group. #*p* < 0.01 versus Sham_Old and I/R_Old; ##*p* < 0.05 versus Sham_Old, *p* < 0.001 versus I/R_Young, *p* < 0.0001 versus I/R_Old; ^*p* <0.01 versus Sham_Old, *p* < 0.0001 versus I/R_Young and I/R_Old; ^^*p* <0.05 versus I/R_Young and *p* < 0.01 versus I/R_Old; §*p* < 0.05 versus Sham_Old and I/R_Old; §§*p* < 0.05 versus I/R_Young, *p* < 0.01 versus I/R_Old; &*p* < 0.05 versus I/R_Young, *p* < 0.0001 versus I/R_Old; &&*p* < 0.05 versus Sham_Old and I/R_Young; @*p* < 0.01 versus I/R_Young, *p* < 0.0001 versus I/R_Old; @@*p* < 0.01 versus Sham_Old, *p* < 0.05 versus I/R_Young. *p < 0.05 between groups; **p < 0.01 between groups; ***p < 0.001 between groups; ****p < 0.0001 between groups. Linear mixed effect model with pair‐wise comparisons between groups at each time points using Tukey's multiple test correction method or one‐way ANOVA with Tukey's multiple test correction method were used between groups

To study the effects of myocardial ischemic injury during aging, both young (3–4 month old) and aged (22 month old) mice underwent surgical I/R injury or sham operation (Study design in Figure 1a). Mortality rate was similar between ischemic groups: 20% mortality for the I/R_Young group (exclusively pre‐operative mortality as three out 15 mice initially included in the study died during surgery or within 24 h post‐surgery) and 23.5% mortality for the I/R_Old group (three mice died during surgery or within 24 h post‐surgery while one mouse died at 8 days post‐ischemic injury, out 17 mice initially included in the study). Sham mice from both young and old groups showed 100% survival rate post‐surgery and throughout the whole duration of the study.

Serial measurements of cardiac function were collected for all mice at baseline (before surgery) as well as at 1, 4, and 8 weeks post‐surgery using standard echocardiography (Table S1). Ejection fraction (EF) was calculated using modified the Simpson method (single plane) and evaluated at baseline and at 1, 4, and 8 weeks post‐surgery. Speckle‐tracking‐based strain echocardiography (Echo strain) was used to evaluate contractility, relaxation, and dyssynchrony in the radial and longitudinal axes at the end of the study.

When we analyzed LV systolic function in terms of EF, and LV dilatation in terms of LV internal diameter in diastole (LVIDd), we found a significant time × group interaction at the *α* = 0.05 level, suggesting that EF and LVIDd changes over time were significantly different among groups. In addition, we performed pair‐wise comparisons between groups at each time point (information and statistics in Table S2). EF was reduced while LVIDd was increased in aged groups when compared to young groups at baseline (Figure 1b,c; information and statistics in Table S2). At the end of our study (8 weeks post‐surgery), LV function was impaired in Sham_Old mice compared to Sham_Young mice (Figure 1b,c and Table S2): EF was 22% lower (mean difference [mean diff.] = −12.9; *p* < 0.01) while LV internal diameter in diastole (LVIDd) was 12% higher (mean diff. = 0.43; *p* = 0.063) in Sham_Old compared to Sham_Young. As expected, at 1, 4, and 8 weeks post‐ischemic injury, LV EF was reduced while LVIDd was increased in I/R_Young mice compared to Sham_Young animals. Specifically, EF was 30% lower (mean diff. = −17.7; *p* < 0.0001) while LVIDd was 16% higher (mean diff. = 0.56; *p* < 0.01) in I/R_Young mice compared to Sham_Young animals at the end of our study. Old mice subjected to ischemic injury showed incremental LV dilatation over the time when compared to I/R_Young mice at 8 weeks post‐ischemic injury (mean diff. = 0.44 and *p* < 0.05) while LV contractility in terms of EF was similar between these groups at 8 weeks post‐ischemic injury (mean diff. = −3.97 and *p* = 0.66) (Figure 1b,c and Table S2).

Interestingly, the I/R_Young group showed earlier impairment in LV function versus I/R_Old group when post‐surgery data were compared to baseline levels. In particular, when we evaluated % change in echocardiographic parameters at 1, 4, and 8 weeks post‐injury compared to baseline we found: (1) I/R_Young showing 17%, 14% and 18% increase in LVIDd while 27%, 34% and 28% decrease in EF, respectively, at 1, 4, and 8 weeks post‐ischemic injury compared to baseline values; (2) I/R_Old mice showing 4%, 10% and 13% increase in LVIDd while 10%, 20% and 18% decrease in EF, respectively, at 1, 4, and 8 weeks post‐ischemic injury compared to baseline values. Figure S1 shows representative B‐mode and M‐mode images obtained from parasternal short‐axis view in all groups at the end of the study.

LV diastolic function was evaluated with standard echocardiography coupled with pulsed wave (PW) Doppler technique at the end of the study. We performed one‐way ANOVA analysis to test any statistically significant difference between groups in the diastolic parameters. In particular, IVRT (F‐value: 9.17), IVRT/RR (F‐value: 11.82), and E wave (F‐value: 7.13) showed a significant difference among means while E/A ratio (F‐value: 1.23) and A wave (F‐value: 1.41) did not show a significant difference (Figure 1d–f and Figure S2A,B). Tukey's post hoc analysis displayed differences between groups in some of the diastolic parameters. Isovolumetric relaxation time (IVRT) and IVRT normalized for RR interval (IVRT/RR) were significantly prolonged (1.7‐ and 1.8‐fold increased, respectively; *p* < 0.01 and *p* < 0.001, respectively) while E wave was 1.3‐fold reduced (*p* = 0.12) in Sham_Old mice compared to Sham_Young mice, indicating impaired LV relaxation (Figure 1d,e and Figure S2A). Both post‐injury groups and the Sham_Old group showed similar impairment in diastolic function according to IVRT, IVRT/RR, and E wave values (Figure 1d,e and Figure S2A). Specifically, IVRT and IVRT/RR were significantly prolonged (1.5‐ and 1.7‐fold increased, respectively; *p* < 0.05 and *p* < 0.01, respectively) while E wave was 1.4‐fold reduced (*p* < 0.05) in I/R_Young mice compared to Sham_Young mice; IVRT and IVRT/RR were significantly prolonged (1.8‐ and 2.1‐fold increased, respectively; *p* < 0.001 and *p* < 0.0001, respectively) while E wave was 1.8‐fold reduced (*p* < 0.001) in I/R_Old mice compared to Sham_Young mice. IVRT, IVRT/RR and E wave were similar between I/R_Young and I/R_Old (*p* = 0.14, 0.14 and 0.40, respectively).

The E/A ratio did not show significant differences between groups (Figure 1f). This is not surprising as the E/A ratio is not a standardized parameter in mice and its widely varying spectrum may correspond to different grades of diastolic dysfunction within the same study group (de Lucia et al., 2019).

To assess for cardiac hypertrophy, we evaluated heart weight (HW) and HW to body weight ratios (HW/BW) at the end of the study in all groups. Specifically, we tested with one‐way ANOVA whether there was an overall difference among means. Statistical difference between groups was found in HW/BW (*F*‐value: 6.72) and HW (*F*‐value: 12.86) but not in BW (*F*‐value: 0.94) (Figure 1g and Figure S2C,D). Multiple comparisons test showed that HW and HW/BW were increased in ischemic hearts compared to age‐matched sham hearts: *p* = 0.18, mean diff. = 0.017 and *p* = 0.12, mean diff. = 0.65, respectively, when I/R_Young versus Sham_Young groups were compared; *p* < 0.01, mean diff. = 0.032 and *p* < 0.05, mean diff. = 0.78 when I/R_Old versus Sham_Old were compared (Figure 1g and Figure S2C). Of note, HW and HW/BW from I/R_Old mice were further augmented (18% and 11% higher, respectively) compared to I/R_Young mice (*p* < 0.01 and *p* = 0.13, respectively) (Figure 1g and Figure S2C).

**Figure 2:**
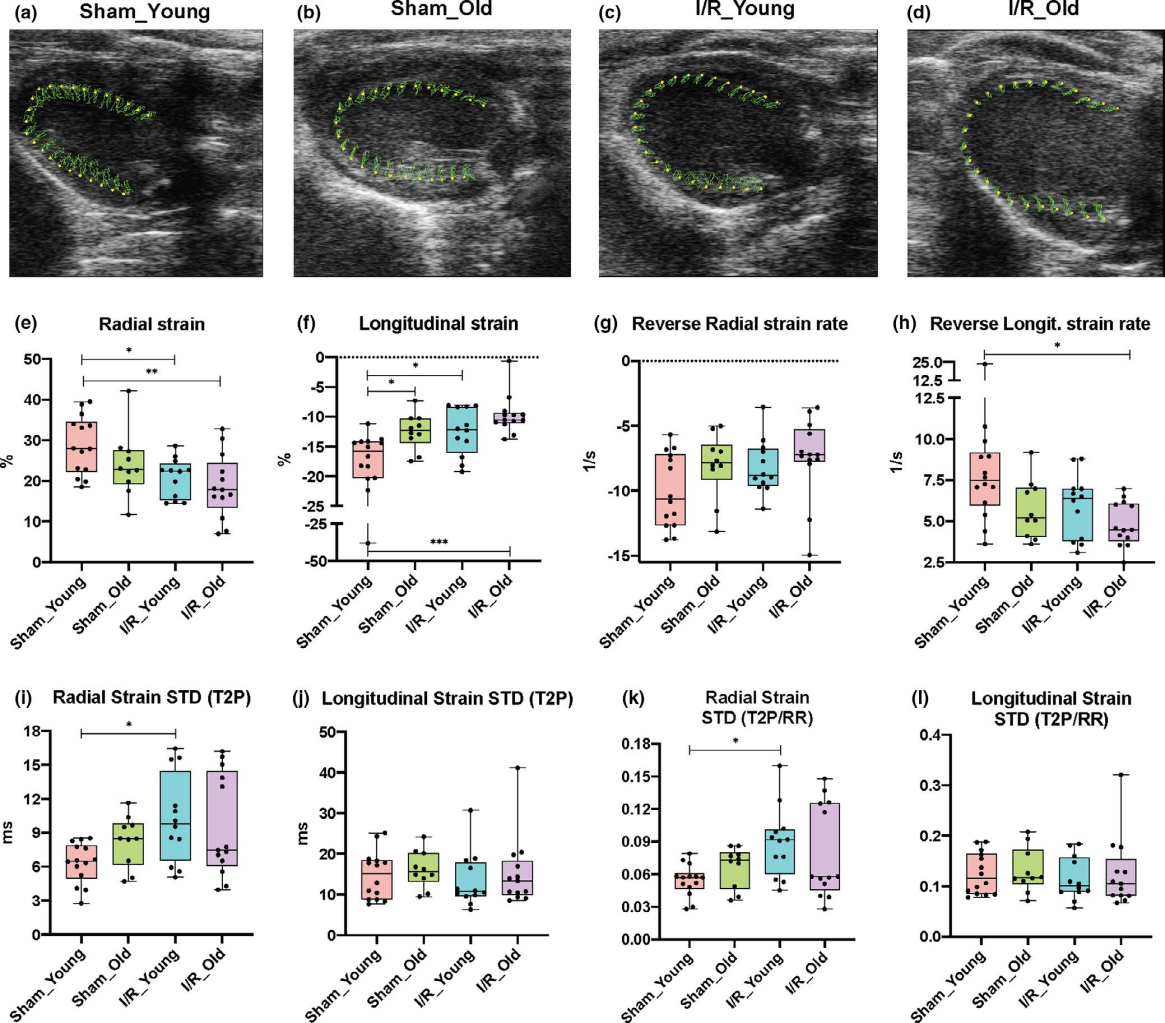
Effects of I/R injury on left ventricular strain and dyssynchrony during aging. Representative pictures of left ventricular deformation (green lines) during the cardiac cycle along the radial and longitudinal axes (parasternal long‐axis view) at 8‐week post‐surgery for each study group: Sham_Young (a), Sham_Old (b), I/R_Young (c) and I/R_Old (d). Radial (e) and longitudinal (f) strain as well as reverse radial (g) and longitudinal (h) strain rate have been measured in Sham and I/R groups. LV dyssynchrony has been assessed: radial and longitudinal standard deviation (STD) of time‐to‐peak (T2P) (I and J, respectively) and STD of T2P/RR ratio (K and L, respectively) have been measured. *n* = 10–14 per group. *p < 0.05 between groups; **p < 0.01 between groups. One‐way ANOVA with Tukey's multiple test correction method was used between groups

Next, advanced measures of cardiac contractility such as radial and longitudinal myocardial strain, were performed using parasternal long‐axis views. Representative images of B‐mode traces obtained with speckle‐tracking analysis are shown in Figure 2a–d. In these images, the green lines represent the path of specific speckles of LV endocardium during the cardiac cycle. Overall difference among groups in radial and longitudinal strain as well as radial and longitudinal strain rate was tested with one‐way ANOVA. Significant difference was found among means for radial strain (*F*‐value: 5.21), longitudinal strain (*F*‐value: 7.65), radial strain rate (*F*‐value: 7.63), and longitudinal strain rate (*F*‐value: 6.13). In line with EF data on systolic function, multiple comparisons test demonstrated that both radial and longitudinal (mainly) strain were deteriorated in Sham_Old mice compared to Sham_Young mice (*p* = 0.37, mean diff. = −4.809 for the radial strain while *p* < 0.05, mean diff. = 5.483 for the longitudinal strain) as well as in I/R_Young to Sham_Young mice (*p* < 0.05, mean diff. = −7.99 for the radial strain while *p* < 0.05, mean diff. = 5.41 for the longitudinal strain) and I/R_Old compared to Sham_Young mice (*p* < 0.01, mean diff. = −10.17 for the radial strain while *p* < 0.001, mean diff. = 8.24 for the longitudinal strain) (Figure 2e,f). Of note, our data showed similar impairment between the injured groups in terms of radial and longitudinal contractility (*p* = 0.87, mean diff. = 2.18 and *p* = 0.42, mean diff. = 2.83, respectively) at the end of the study (Figure 2e,f). Radial and longitudinal strain rate were similarly reduced in Sham_Old, I/R_Young and I/R_Old groups compared to Sham_Young group (*p* = 0.27, *p* < 0.01 and *p* < 0.001, respectively, for the radial strain rate while *p* = 0.07, *p* = 0.30 and *p* < 0.001, respectively, for the longitudinal strain rate) (Figure S3A,B). Importantly, these parameters were comparably impaired in old and young mice within the ischemic groups (*p* = 0.74 for radial strain rate and *p* = 0.11 for longitudinal strain rate) (Figure S3A,B).

Reverse longitudinal strain rate, which reflects myocardial deformation in diastole on the longitudinal axis, was significantly different between groups at the end of the study (one‐way Anova *F*‐value: 3.81). In particular, pair‐wise comparisons between groups revealed that reverse longitudinal strain rate was decreased in the ischemic groups and the Sham_Old group compared to Sham_Young mice with I/R_Old mice showing the greatest impairment (*p* < 0.05 and mean diff. = 3.65 compared to the Sham_Young group) (Figure 2h). Reverse radial strain rate, reverse longitudinal strain, and reverse radial strain were also tested and were found not different between groups (one‐way ANOVA *F*‐values were 2.23, 0.22, 0.85, respectively) (Figure 2g and Figure S3C,D).

LV dyssynchrony was assessed with Echo strain. Overall difference among groups for the following parameters was tested: time‐to‐peak variation defined as the standard deviation (STD) of T2P over 6 LV segments, STD of T2P normalized to RR interval (T2P/RR) and maximum T2P delay between the earliest and the latest segment (Figure 2i–l and Figure S3E,F). Radial strain STD of T2P (one‐way ANOVA F‐value: 3.48) and radial strain STD of T2P/RR (one‐way ANOVA *F*‐value: 3.41) but not radial strain maximum T2P delay (one‐way ANOVA F‐value: 2.59) were different between groups (Figure 2i,k and Figure S3E). Injured groups showed similar increment in LV dyssynchrony in the radial axis compared to the Sham_Young group as demonstrated by the increase in radial STD of T2P (*p* < 0.05, mean diff. = 3.92 for “I/R_Young vs. Sham_Young” while *p* = 0.077, mean diff. = 3.20 for "I/R_Old vs. Sham_Young") and radial STD of T2P/RR (*p* < 0.05, mean diff. = 0.035 for “I/R_Young vs. Sham_Young” while *p* = 0.13, mean diff. = 0.026 for "I/R_Old vs. Sham_Young") at multiple comparisons test (Figure 2i,k). LV dyssynchrony in the longitudinal axis was not altered by age or cardiac injury (Figure 2j,l and Figure S3F).

### 2.2 Global plasma metabolomic profiling

**Figure 3:**
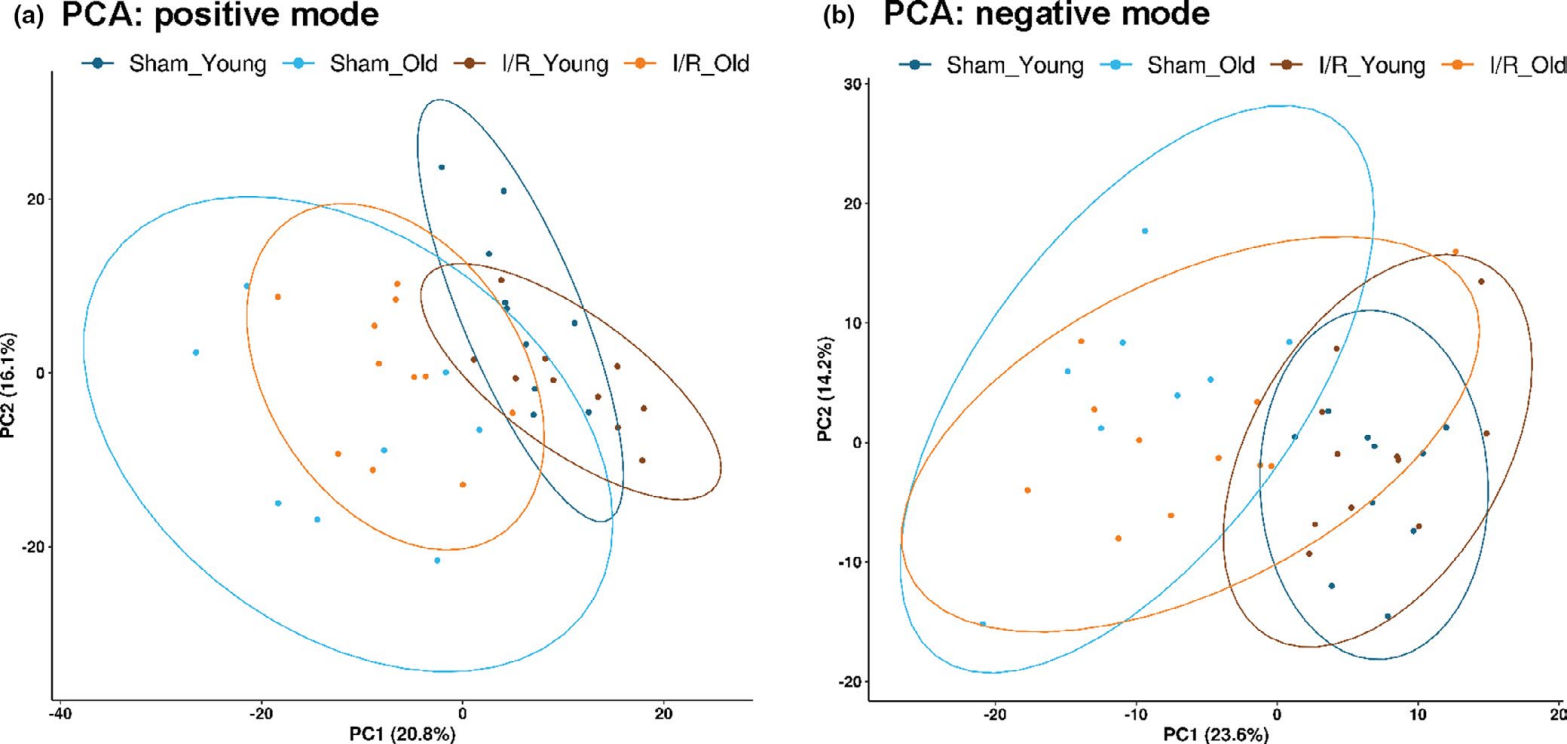
Plasma metabolomic profile in young and old mice subjected to I/R injury or sham operation—Principal component analysis: Principal component analysis (PCA) was performed for metabolic features detected by LC/MS method in both positive (a) and negative (b) ion modes. We generated a static 2D PCA (PC1 vs. PC2) with 0.9 data ellipses. The percentage of the variance explained by each PC is shown in parentheses

**Figure 4:**
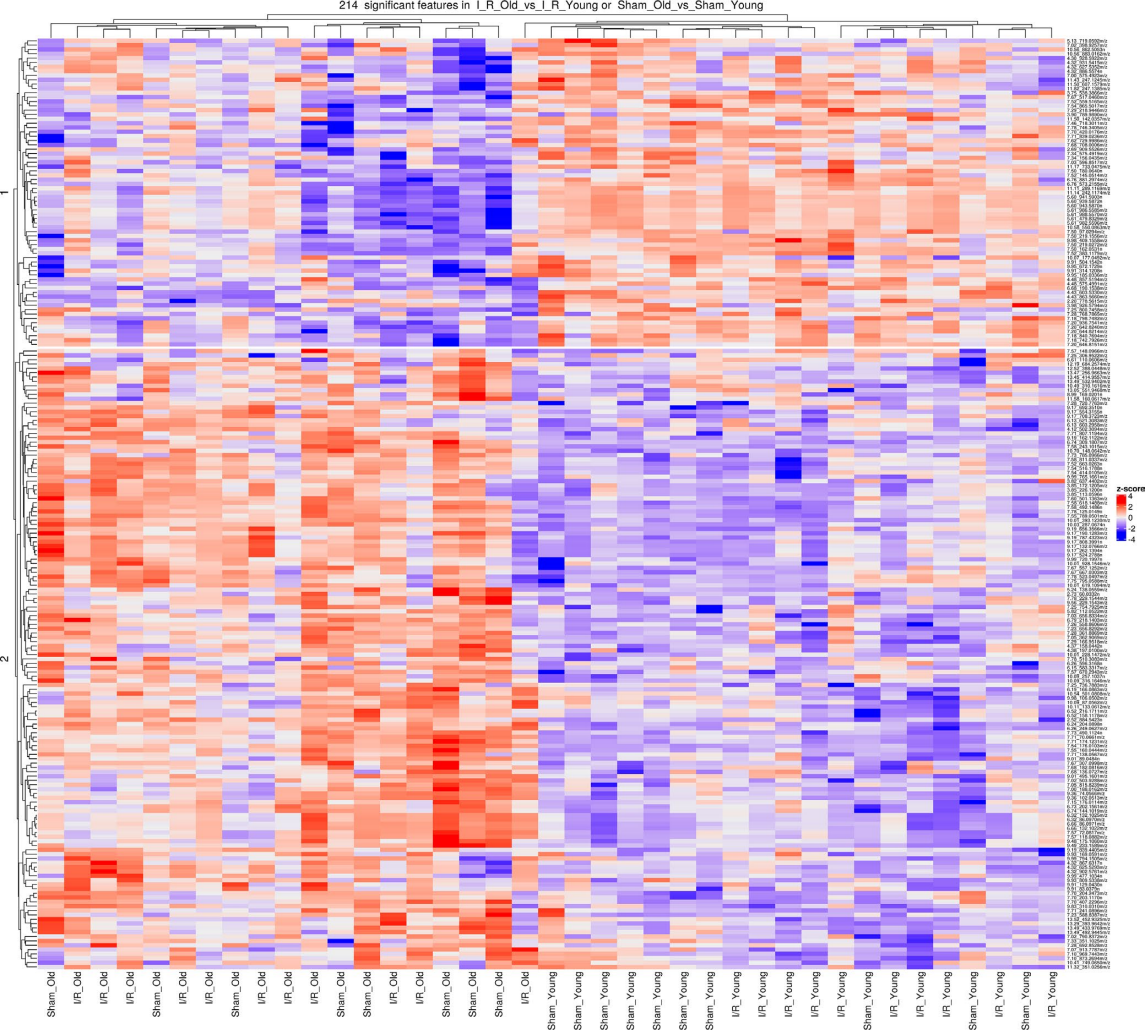
Plasma metabolomic profile in young and old mice subjected to I/R injury or sham operation – Heatmap, positive mode: Heatmap and hierarchical clustering analysis generated for the metabolites (identified in positive mode) that have a significant difference in either “Sham_Old vs Sham_Young” or “I/R_Old vs I/R_Young,” We computed a z‐score, where we adjusted the data by feature, to have a mean of zero and a standard deviation of 1. The resulting heatmap presents the features (reported on the right side) in rows and samples in columns, both of which are clustered

**Figure 5:**
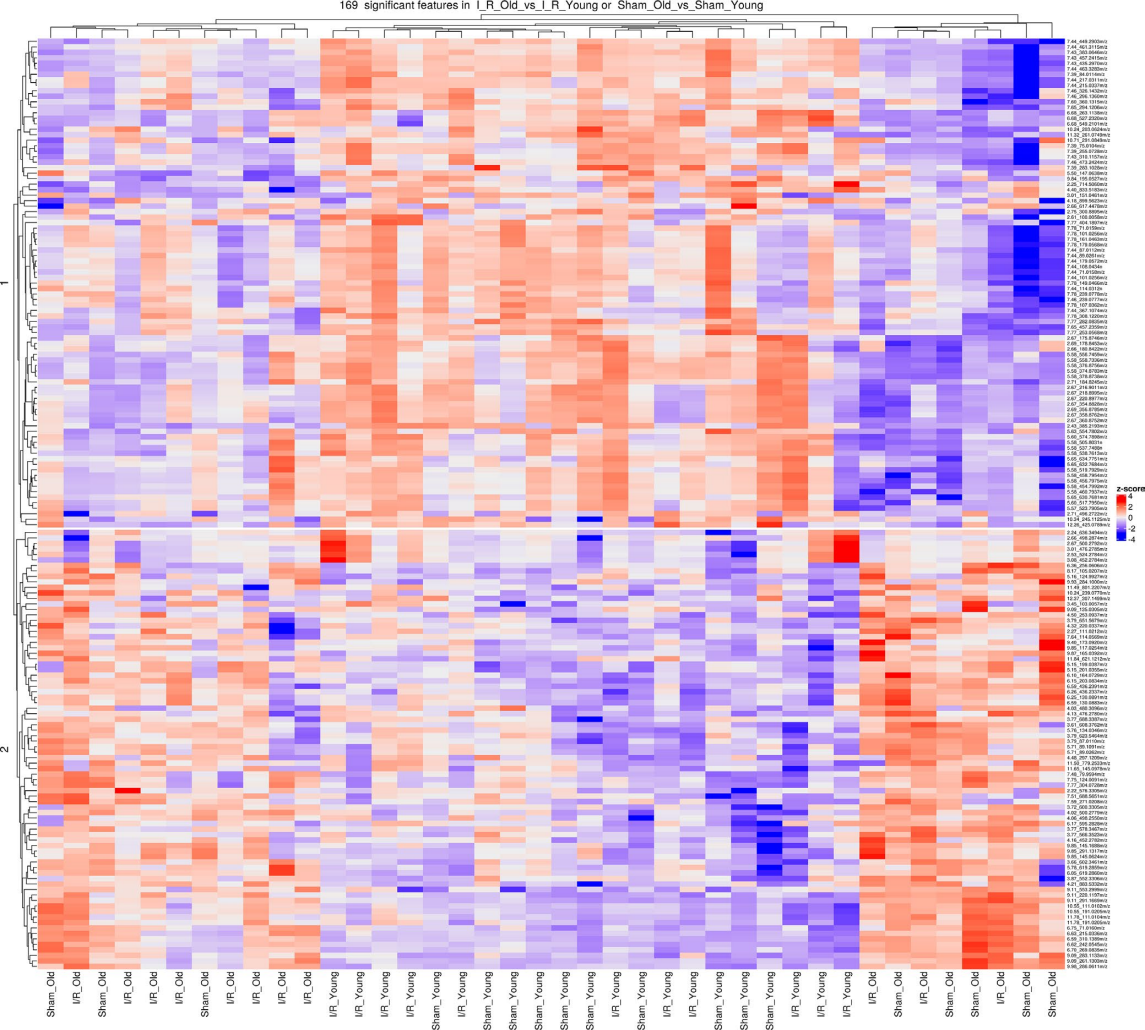
Plasma metabolomic profile in young and old mice subjected to I/R injury or sham operation – Heatmap, negative mode: Heatmap and hierarchical clustering analysis generated for the metabolites (identified in negative mode) that have a significant difference in either “Sham_Old vs Sham_Young” or “I/R_Old vs I/R_Young.” We computed a z‐score, where we adjusted the data by feature, to have a mean of zero and a standard deviation of 1. The resulting heatmap presents the features (reported on the right side) in rows and samples in columns, both of which are clustered

Plasma metabolomic alterations have been reported during aging and heart disease in both humans and animal models (Bunning et al., 2020; Houtkooper et al., 2011; Johnson et al., 2018; McGarrah et al., 2018). Accordingly, we investigated whether age and myocardial ischemic injury may induce a combined effect on circulating metabolites. To do this, we collected plasma samples from young and old mice subjected to I/R injury or sham operation (Study design, Figure 1a) and used a global (untargeted) high‐resolution metabolomics approach focused on aqueous metabolites. We detected 1386 and 891 metabolite features in positive ionization mode and negative ionization mode, respectively, after reducing isotope and adduct peaks (Appendix S1 and S2). After data pretreatment and filtering, the total metabolite features resulted in 603 features in positive ionization mode and 433 features in negative ionization mode (Appendix S3 and S4). To identify potential effects of age and surgical injury on the metabolomic profiles, we first performed an unsupervised principal component analysis (PCA) on metabolite features in positive and negative modes. We found that PC1 and PC2, which together capture the greatest variance across the dataset (36.9% in positive mode and 37.8% in negative mode), clearly separated the groups by age (Figure 3). Then, we generated heatmaps for both positive and negative modes reporting the features (IDs on the right side) that had a significant difference in either “I/R_Old versus I/R_Young” or “Sham_Old versus Sham_Young” (Figure 4 for positive mode while Figure 5 for negative mode). Interestingly, there was a group of features decreasing with age (cluster 1) with another group increasing with age (cluster 2); clustering was evident in both positive and negative modes (Figures 4 and 5, respectively). More information on each feature (including clustering, mummichog's putative metabolite IDs and pathway involved) is shown in Appendix S5 and S6 (respectively for positive and negative modes).

We next fit a linear model to the normalized and imputed data in order to detect group differences, while controlling for the estimated surrogate variables (SVs) as covariates in our model. Then, we selected features with a false discovery rate (FDR) of 10%. In positive mode data, we found (a) 181 features showing significant changes in their log_2_‐abundance levels between Sham_Old versus Sham_Young, (b) 128 features showing significant changes between I/R_Old and I/R_Young, and (c) only one feature resulted significantly different when we tested the interaction between surgery type and age (Appendix S7). In negative mode data, we found (a) 157 features showing significant changes in their levels between Sham_Old versus Sham_Young, (b) 89 features showing significant changes between I/R_Old and I/R_Young, and (c) five features showing significant changes between I/R_Young and Sham_Young (Appendix S8). In positive mode data, 95 features were in common between “Sham_Old vs Sham_Young” and “I/R_Old and I/R_Young” comparisons while in negative mode 77 features were in common between “Sham_Old vs Sham_Young” and “I/R_Old and I/R_Young” comparisons (Figure S4).

**Figure 6:**
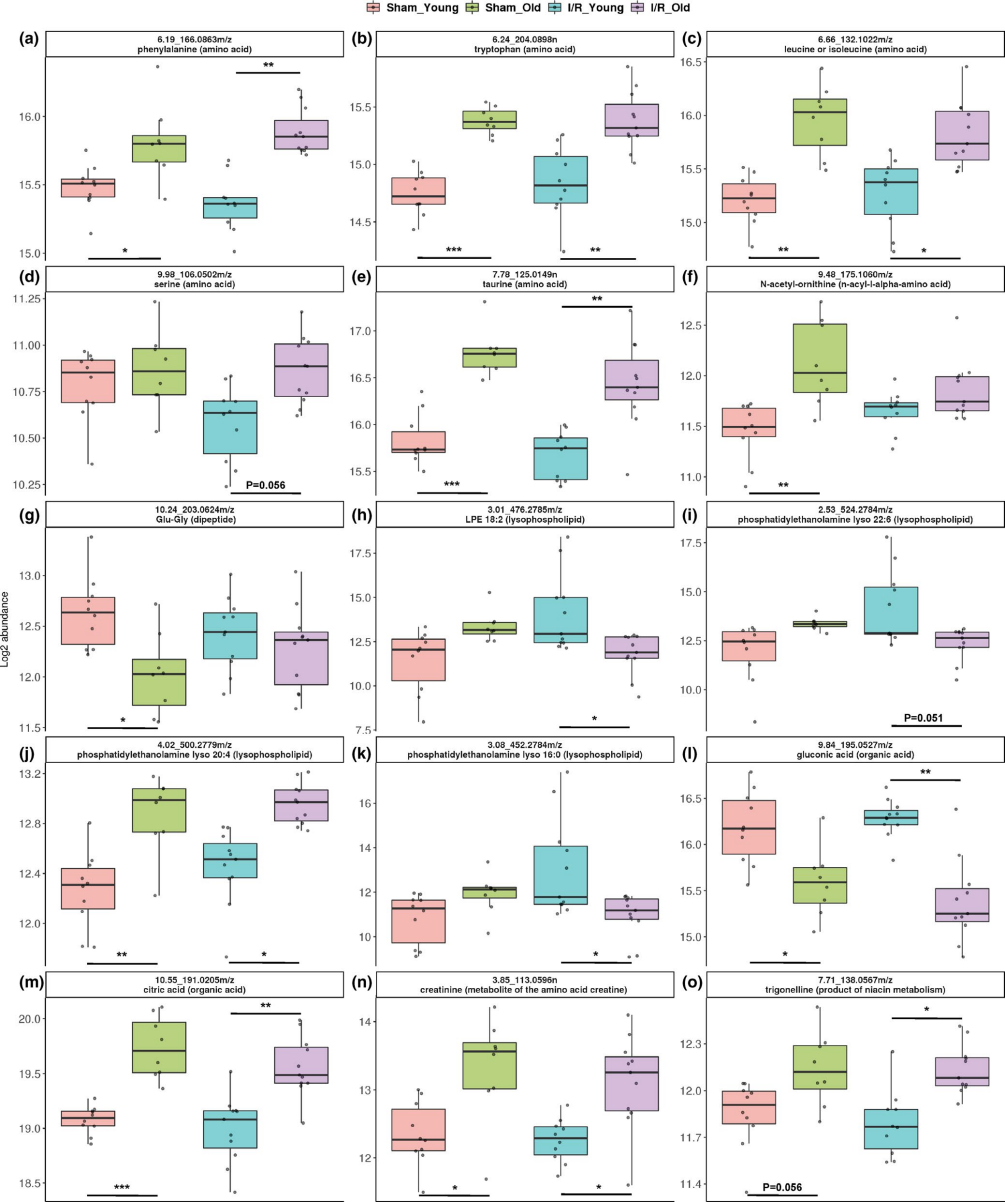
Plasma metabolites/mass features differently abundant in young and old mice subjected to I/R injury or sham operation. Boxplots of mass features corresponding to metabolite ID and showing a significant difference in “Sham_Old vs Sham_Young” and “I/R Old vs I/R_Young.” The compound identification proceeded by MS/MS analysis: a: phenylalanine; b: tryptophan; c: leucine or isoleucine; d: serine; e: taurine; f: N‐acetyl‐ornithine; g: Glu‐Gly; h: LPE (18:2); i: phosphatidylethanolamine lyso 22:6; j: phosphatidylethanolamine lyso 20:4; k: phosphatidylethanolamine lyso 16:0; l: gluconic acid; m: citric acid; n: creatinine; o: trigonelline. The biological molecule class of each metabolite has been specified in parenthesis. We have calculated adjusted *p*‐value (FDR using Benjamini–Hochberg procedure) for each of the comparisons: *p < 0.05 between groups; **p < 0.01 between groups; ***p < 0.001 between groups

We have also predicted some metabolites that were differently abundant among groups according to the molecular formulae (Figure 6; Figures S5 and S6; Table S3 and S4). Most of the predicted metabolites were then confirmed with MS/MS analysis (MS/MS spectra in Figure S7).

Of the metabolites that showed age‐specific changes in sham and/or I/R groups, several were amino acids such as phenylalanine, tryptophan, leucine/isoleucine, serine, glutamine, valine, glutamic acid, taurine, N‐acetyl‐ornithine, creatine, N‐iminoethylornithine or amino acid‐derivative such as carnitine and proline betaine or dipeptides such as Glu‐Gly, Lys‐Asp, Pro‐Leu/L,L‐TMAP (Figure 6 and Figure S5; information and statistics in Tables S3 and S4). Of note, several lysophospholipids such as LPE 18.2, phosphatidylethanolamine lyso 22:6, phosphatidylethanolamine lyso 20:4, phosphatidylethanolamine lyso 16:0 and LPA 20:4 were affected by age in sham and/or ischemic groups (Figure 6 and Figure S6; information and statistics in Tables S3 and S4). Other metabolites perturbed by age in sham and/or I/R groups were: gluconic acid, citric acid, creatinine, trigonelline, lactic acid and methylxanthine (Figure 6 and Figure S6; information and statistics in Table S3 and S4). Interestingly, only five features corresponding to phosphatidylethanolamine‐like metabolites and a benzenoid‐like metabolite such as anisic acid or hydroxyphenylacetic acid were differently abundant between I/R_Young versus Sham_Young groups. (Figure S6 and Table S3 and S4).

We used the Mummichog algorithm to test the enrichment of pathways and to generate putative metabolite annotations both in positive and negative modes. Among the pathways significantly different in both “Sham_Old vs Sham_Young” and “I/R_Old vs I/R_Young” comparisons, we observed enrichment for the following pathways: tRNA charging pathway, pyridoxal 5′‐phosphate salvage pathway, adenine and adenosine salvage, glucose/glucose‐1‐phosphate degradation, and galactose degradation I (Leloir pathway) (information and statistics in Tables 1 and 2). Several other pathways involving amino acid, glucide, and nucleic acid metabolism were also affected by age in sham and ischemic groups (information and statistics in Tables 1 and 2). Further, our metabolite prediction with the Mummichog program confirmed numerous amino acids to be differently abundant in the plasma of aged mice of both sham and infarcted groups (Figure S8). Moreover, the bile acid biosynthesis neutral pathway was enriched and taurochenodeoxycholate/taurocholate were predicted to be different when I/R groups were compared (Table 2 and Figure S8).

**Table 1:** Top pathways ‐ positive mode

Sham_Old versus Sham_Young				
Pathways	overlap_size	pathway_size	*p*‐value (raw)	*p*‐value
nicotine degradation II	4	4	0.14074	0.07795
nicotine degradation III	4	4	0.14074	0.07795
pyridoxal 5′‐phosphate salvage pathway	3	3	0.23209	0.17525
tyrosine biosynthesis II	2	2	0.38015	0.43254
catecholamine biosynthesis	2	2	0.38015	0.43254

I/R_Old versus I/R_Young				
Pathways	overlap_size	pathway_size	*p*‐value (raw)	*p*‐value
tRNA charging pathway	7	10	0.10639	0.00278
asparagine biosynthesis I	3	3	0.09624	0.00694
pyridoxal 5′‐phosphate salvage pathway	3	3	0.09624	0.00694
uridine−5′‐phosphate biosynthesis	3	4	0.25596	0.02005
asparagine degradation I	2	2	0.21263	0.03372
glutamine degradation II	2	2	0.21263	0.03372
tryptophan degradation I (via anthranilate)	2	2	0.21263	0.03372
galactose degradation I (Leloir pathway)	2	2	0.21263	0.03372

Note

Enrichment of pathways was tested using Mummichog software. Differences between “Sham_Old vs Sham_Young” and “I/R_Old vs I/R_Young” in positive mode have been shown. An adjusted *p*‐value per pathway is calculated based on the EASE score and cumulative distribution function (Li et al., 2013).

**Table 2:** Top pathways ‐ negative mode

Sham_Old versus Sham_Young				
Pathways	Overlap_size	Pathway_size	*p*‐value (raw)	*p*‐value
tRNA charging pathway	5	8	0.55167	0.01285
sucrose degradation V (mammalian)	3	4	0.44072	0.01328
guanine and guanosine salvage II	2	2	0.33516	0.0188
adenine and adenosine salvage III	2	2	0.33516	0.0188
S‐adenosyl‐L‐methionine cycle II	2	2	0.33516	0.0188
lipoate biosynthesis and incorporation I	2	2	0.33516	0.0188
lipoate biosynthesis and incorporation II	2	2	0.33516	0.0188
glycine betaine degradation	2	2	0.33516	0.0188
arsenate detoxification I (glutaredoxin)	2	2	0.33516	0.0188
sucrose degradation	2	2	0.33516	0.0188
galactose degradation I (Leloir pathway)	2	2	0.33516	0.0188
lactose degradation III	2	2	0.33516	0.0188
melibiose degradation	2	2	0.33516	0.0188
glucose and glucose−1‐phosphate degradation	2	2	0.33516	0.0188
methionine degradation I (to homocysteine)	2	2	0.33516	0.0188

I/R_Old versus I/R_Young				
Pathways	overlap_size	pathway_size	*p*‐value (raw)	*p*‐value
bile acid biosynthesis, neutral pathway	3	4	0.25705	0.00542
adenine and adenosine salvage III	2	2	0.21341	0.01468
glucose and glucose−1‐phosphate degradation	2	2	0.21341	0.01468
L‐ascorbate biosynthesis VI	2	3	0.44714	0.07683
adenosine nucleotides degradation II	2	3	0.44714	0.07683

Note

Enrichment of pathways was tested using Mummichog software. Differences between “Sham_Old vs Sham_Young” and “I/R_Old vs I/R_Young” in negative mode have been shown. An adjusted *p*‐value per pathway is calculated based on the EASE score and cumulative distribution function (Li et al., 2013).

Finally, we evaluated whether there was any correlation between the metabolite data and the cardiac functional data. We specifically tested the correlation of metabolite data and markers of LV systolic function (EF), dilatation (LVIDd), diastolic function (Reverse radial and longitudinal strain rate), and cardiac hypertrophy (HW/BW). We generated PCA plots (after removing estimated SVs) that were color‐coded by cardiac function variables (positive mode in Figure S9 and negative mode in Figure S10). Then, we fitted a linear model to the normalized and imputed data to detect if there was a linear relationship between metabolite abundance and each of the cardiac function variables within each group while controlling for estimated SVs in our model. Results are displayed in Appendix S9: we have included information of all the individual metabolites tested for each of the comparisons. We then selected features with a false discovery rate (FDR) of 10% and found that some features were correlated with the cardiac parameters: mainly within the I/R_Old group and only one feature within the Sham_Young group. We generated graphs of the log2‐abundance of metabolite features versus each of the cardiac function variables for those that were significant (Figures S11 and S12 for positive and negative modes). Of note, the feature “9.87_165.0392 m/z” that correlated with the reverse radial strain rate within the I/R_Old group was identified as methylxanthine.

## 3 DISCUSSION

Heart disease is the leading cause of mortality, hospitalization, and rehospitalization in the United States and worldwide (Benjamin et al., 2019). Projections predict that the prevalence of HF will increase 46% from 2012 to 2030, resulting in >8 million people with this disease syndrome in the United States (Benjamin et al., 2019). Importantly, age is the main risk factor for several disorders including heart disease and, the prevalence and incidence of HF increase dramatically with age. For the 60– 79‐year‐old age group, 6.9% of men and 4.8% of women have HF (Benjamin et al., 2019). Hence, the management of elderly patients with HF (with coronary artery disease and ischemic injury being a common cause) entails great challenge and considerably impacts health care costs (Dharmarajan & Rich, 2017). The aged heart shows several structural, functional and molecular changes that make it more vulnerable and prone to stressors such as myocardial ischemia, hypertension, diabetes, metabolic syndrome, and other comorbidities (Chiao & Rabinovitch, 2015; Dharmarajan & Dunlay, 2016). Aging is an extremely complex process that impairs several systems and biological pathways (Hoffman et al., 2017). Ischemic HF is a multifactorial and systemic disease as well, with several cellular, molecular, and neuro‐hormonal mechanisms being compromised during both the acute and chronic phases (Dharmarajan & Rich, 2017).

Wide metabolic changes in different organs occur during both aging and heart failure and, some of them can be captured with the assessment of circulating metabolites (Houtkooper et al., (2011); Bunning et al., 2020; McGarrah et al., 2018; Johnson et al., 2018; Lee et al., 2014; Zordoky et al., 2015; Chaleckis et al., 2016; Gonzalez‐Freire et al., 2019). However, in the clinical setting, it may be complicated to study whether some of those metabolic alterations were influenced by additional diseases as several older adults especially with heart dysfunction show multiple comorbidities (Dharmarajan & Dunlay, 2016). Plasma metabolomics allows the unbiased profiling of a large panel of circulating metabolites and has become a useful analytical tool to evaluate the pathophysiological mechanisms involved in aging as well as to predict outcomes in patients with heart disease (Bunning et al., 2020; Houtkooper et al., 2011; McGarrah et al., 2018).

The aim of our study was to evaluate the effects of age and ischemic injury on the plasma metabolome in mice, with the goal eliminating the confounding influence of age‐related comorbidities such as diabetes, metabolic syndrome, and chronic kidney disease. The introduction of timely myocardial reperfusion strategies in the recent years has considerably reduced the mortality rate for both young and older adults with acute myocardial infarction (Van de Werf, 2018). Hence, to reproduce the clinical setting of the ischemic heart disease in both young and elderly patients as closely as possible, we chose to study a mouse model of reperfused myocardial infarction (compared to sham operation) in young and old mice (Gao et al., 2010). Survival rate was similar between young and old mice subjected to I/R injury with the majority of deaths occurring within 24 h post‐surgery.

First of all, we performed a complete assessment of cardiac performance (including LV systolic and diastolic function as well as synchronicity) in young and old mice with and without cardiac ischemic injury. As expected, I/R injury dramatically worsened cardiac performance and increased cardiac hypertrophy in young mice compared to the Sham_Young group. Both LV systolic (in terms of EF) and diastolic function (as suggested by increased IVRT and IVRT/RR as well as decreased E wave) were impaired and LV dimensions were enlarged in I/R_Young mice compared to control Sham_Young mice. We also took advantage of a novel and interesting technique, Echo strain, in order to accurately evaluate LV systolic/diastolic function and dyssynchrony. LV strain evaluation has been shown to be reproducible and highly sensitive both in patients with heart disease and in animal models (de Lucia et al., (2019); Potter & Marwick, 2018). We found that LV contractility was impaired in both radial and longitudinal axis, while relaxation was mainly compromised in longitudinal axis in I/R_Young mice compared to Sham_Young mice. Intra‐ventricular synchronicity has been shown as a crucial prognostic factor in patients with heart disease and we confirmed that the I/R_Young group had increased LV dyssynchrony in the radial axis compared to young controls (Cai and Ahmad, (2015)). As previously demonstrated, aged sham mice displayed impairment in LV systolic function (in terms of EF and predominantly longitudinal strain) and diastolic function (augmented IVRT and IVRT/RR as well as decreased reverse longitudinal strain rate) when compared to Sham_Young mice (de Lucia et al., (2019)). Interestingly, we found that ischemic aged mice showed further LV dilatation and cardiac hypertrophy when compared to failing young and Sham_Old groups. LV systolic and diastolic function as well as dyssynchrony were not significantly further impaired in failing old mice compared to aged mice and were similarly compromised in young and old mice subjected to I/R injury.

Our data are not completely in line with previous reports showing increased susceptibility of the aged heart to reperfused myocardial ischemia in animal models (Bujak et al., (2008); Du et al., 2017; Quan et al., 2017; Azhar et al., 1999). Several reasons may justify the differences between our findings and previously published studies. First of all, in our study we have used a novel, minimally invasive surgical technique to induce I/R injury that may be less harmful in the aged mice when compared to the standard I/R technique (Gao et al., 2010). In fact, our surgical approach has been shown to display lower acute local and systemic inflammation and easier postsurgical recovery. Both these factors are expected to be crucial for post‐injury LV response and remodeling especially in frail, aged mice (Gao et al., 2010). In addition, the time of LAD coronary artery occlusion may have contributed to the dissimilarities with other studies as our model consisted of 30 min of occlusion while other studies reported 45–60 min (Azhar et al., 1999; Bujak et al., 2008; Du et al., 2017; Quan et al., 2017). Importantly, we consider our model close to the real clinical setting as timely reperfusion (fibrinolysis or primary percutaneous coronary intervention) is highly recommended (Steg et al., 2012). Moreover, we followed our groups longer (8 weeks) than the other studies (24 h, 7 days or 3 weeks) and evaluated only male mice (to reduce the gender‐related bias) while other studies did not report the gender (only Azhar et al. acknowledged male use) (Azhar et al., 1999; Bujak et al., 2008; Du et al., 2017; Quan et al., 2017). Of note, worsened cardiac function and outcomes of elderly patients with ischemic HF compared to young HF patients in the clinical practice are strongly influenced by concomitant comorbidities and increased frailty (Dharmarajan & Dunlay, 2016; Dharmarajan & Rich, 2017).

To pursue the primary purpose of our study, we used an untargeted approach to characterize plasma metabolomic alterations in old and young mice subjected to ischemic injury or sham operation. We discovered several circulating metabolites to be differently abundant in young versus old mice of both sham and ischemic groups. Of note, “Sham_Old mice versus to Sham_Young” and “I/R_Old mice versus to I/R_Young” comparisons shared numerous metabolites, strongly suggesting aged groups to have shared pattern/clusters and ischemic injury to have not played an additive role. In fact, only one feature was found in our statistical analysis when we tested the interaction between age and surgical type. Of note, so far the ID and function of this feature is still unknown.

We confirmed some findings from previous studies in humans and animal models showing marked changes in several amino acids (increased essential amino acids phenylalanine, tryptophan, valine and leucine/isoleucine and non‐essential glutamic acid) as well as phospholipids, creatine, and xanthine plasma levels with age (Bunning et al., 2020; Chaleckis et al., 2016; Gonzalez‐Freire et al., 2019; Houtkooper et al., 2011; Johnson et al., 2018; Lee et al., 2014; Rist et al., 2017; Seo et al., 2016). The Mummichog analysis also suggested several pathways involving amino acid, glucide, and nucleic acid metabolism to be impacted by age in both sham and ischemic groups. Furthermore, it is not surprising that “tRNA charging pathway” (predicted as several amino acids were different between old and young groups) was the pathway most altered by age in both ischemic and control group as tRNAs are regulators of key biological processes such as protein biosynthesis and gene expression (Raina & Ibba, 2014). Of note, cellular and circulating amino acids play several roles both in young and old age such as fueling the tricarboxylic acid cycle (the Krebs cycle) in mitochondria, an organelle known to be affected by aging and by I/R injury (Ham and Raju, (2017); Martínez‐Reyes & Chandel, 2020; Sharma & Ramanathan, 2020). Impaired circulating amino acid levels may be strictly related to the altered mitochondrial function in aged mice (Ham and Raju, (2017); Martínez‐Reyes & Chandel, 2020; Sharma & Ramanathan, 2020). Pyridoxal 5′‐phosphate savage pathway, essential cofactor of numerous metabolic enzymes and mainly involved in amino acid metabolism, was also changed with age in our study (Bode and Berg, 1991). Bile acid biosynthesis pathway was affected by age only in the ischemic group. In addition, both taurocholate and taurodeoxycholate were predicted as differentially expressed in aged ischemic mice and have been previously associated with human longevity (Cheng et al., 2015). Bile acids have been recently found to control not only cholesterol homeostasis but also to regulate glucose and energy metabolism (Cheng et al., 2015). Taurine has been previously associated with gender differentiation in humans as well as with primary bile acid biosynthesis and was increased in both aged groups in our study (Jové et al., 2016). Phenylalanine was increased in the aged failing group and has been previously included as part of a panel (together with histidine and ornithine) to identify patients with HF at stage C (ACC/AHA HF classification), particularly in the elderly sub‐group (Wang et al., 2018). It is also not surprising that aged groups showed higher plasma creatinine levels strongly suggesting age‐associated loss of kidney function. Circulating lactic acid was also increased in the aged compared to the young groups. This may be mainly due to its impaired liver rather than muscle metabolism, as previously shown (Houtkooper et al., 2011).

Only five mass features corresponding to two metabolites were differently abundant in I/R_Young mice compared to Sham_Young mice. One of them has been predicted as “benzenoid, anisic acid or hydroxyphenylacetic acid” and was reduced in both ischemic groups. 3‐hydroxyphenylacetic acid is an antioxidant while 4‐hydroxyphenylacetic acid has been shown to attenuate inflammation and edema in lung injury in rats (Liu et al., (2014)). In addition, quercetin exerts its cardio‐protective effects at least in part via its metabolite 3‐hydroxyphenylacetic acid, in I/R injury (Dong et al., (2018); Dabeek & Marra, 2019). The other features were predicted as lysophosphatidylethanolamines and were increased in ischemic young mice compared to age‐matched sham mice, in line with previous human study (Park et al., 2015).

When we evaluated whether there were any correlations between the metabolite data and the cardiac functional data in our study, we found some features to be correlated to LV systolic and diastolic parameters within the I/R_Old group. Interestingly, one feature identified as methylxanthine, was correlated to the reverse radial strain rate within the I/R_Old group. Further studies will be necessary to classify the other features and understand if our correlations are also confirmed in elderly patients with heart disease.

As several studies showed a peculiar metabolic signature in patients with CAD, myocardial ischemia or HF and, a large part of those patients are older adults, we expected to find in our study a specific circulating metabolomic pattern in the ischemic groups (Lanfear et al., 2017; McGarrah et al., 2018). We found instead that aging was the main factor differentiating plasma metabolites in both sham and injured groups. We may explain the discrepancy between our study and the clinical setting as patients with heart disease, particularly an older sub‐group, often display multiple comorbidities including diabetes, metabolic syndrome, endocrine diseases, and chronic kidney disease that can considerably impact plasma metabolic profile (Madhavan et al., 2018; Tromp et al., 2020). In this regard, previous studies showed that diabetes, obesity, and chronic kidney disease could significantly influence several metabolic pathways as well as circulating metabolomic signature in healthy controls and patients with heart disease (Cirulli et al., 2019; Lanfear et al., 2017; Rhee, 2015; Tromp et al., 2020). However, we should not completely exclude that changes related to species (humans vs. mice) or experimental procedures may explain at least in part the differences between our data and previous clinical studies.

In conclusion, using a minimally invasive model of reperfused myocardial ischemia injury, we have found that ischemic injury may not drastically reduce cardiac function in aged compared to young mice. Furthermore, our study shows for the first time that ischemic injury did not significantly affect plasma metabolomic profile in young or old mice. Further studies in humans and animal models will clarify whether, and how, different comorbidities affect cardiac function and plasma metabolomic signature in elderly patients/aged animals with ischemic heart disease.

## 4 EXPERIMENTAL PROCEDURES

### 4.1 Experimental animals

All animal procedures were performed in accordance with the guidelines of the Institutional Animal Care and Use Committee of Temple University School of Medicine. Young (3–4 month old) and old (22 month old) C57BL/6 male mice were subjected to I/R injury or sham operation as previously described (Gao et al., 2010).

Plasma samples were collected at the end of the study (8 weeks post‐surgery) between 10 a.m. and 12 p.m. in EDTA‐coated tubes (Sarstedt). Then the tubes were immediately mixed by inversion to ensure complete mixing with anticoagulant. This was followed by centrifugation at 3000 RPM at 4°C for 10 min and subsequent aliquoting in tubes. Samples were snap frozen and then stores at −80°C.

### 4.2 Echocardiography and gravimetric analysis

To assess cardiac structure and function, transthoracic echocardiography was performed using a VisualSonics Vevo 2100 system (VisualSonics, Toronto, Ontario, Canada) with a MS400 (30‐MHz centerline frequency) probe, as previously described (de Lucia et al., 2019). In brief, mice were anesthetized with isoflurane (induction 3% and maintenance 1.5–2%) and hair was removed from the chest. Then, mice were placed in a supine position on a heated table (core temperature was maintained at 37°C) with embedded ECG leads.

B‐and M‐mode images were acquired from a parasternal short‐axis view to evaluate LV FS, LVIDd, LVIDs, volume at diastole and systole, anterior wall in diastole and systole, posterior wall in diastole and systole and heart rate (de Lucia et al., 2019).

Diastolic function was evaluated using conventional echocardiography coupled with PW Doppler techniques (de Lucia et al., 2019). From an apical long‐axis view, transmitral inflow velocities were recorded by setting the sample volume in the mitral orifice close to the tip of the mitral leaflets. From the PW spectral waveforms, we measured the peak early‐ and late‐diastolic transmitral velocities (E and A waves) to obtain the E/A ratio, and IVRT.

Echocardiographic speckle‐tracking analyses were performed on parasternal long‐axis B‐mode loops using a VisualSonics Vevo 2100 system (VisualSonics, Toronto, Ontario, Canada), as previously described (de Lucia et al., 2019; Weinheimer et al., 2018). All images were acquired and then analyzed using the Vevo Software (Vevo LAB 3.1.1) under their coded numbers in a blinded fashion; then, the code was broken and statistical analysis was performed. EF has been calculated (with the Vevo LAB Software) using the modified Simpson method (single plane): The LV volume is calculated from the computed border by the modified Simpson method using 64 equispaced disks from the mitral plane to the apex. The ejection fraction is then calculated as EF = 100 × (Vd − Vs)/Vd, where the end‐systolic and end‐diastolic volumes (Vs and Vd, respectively) are computed as the minimum and maximum values in the averaged R‐R interval (Otto, 2013; Pedrizzetti et al., 2014).

LV endocardial strain and strain rate were calculated with Vevo Software as an average of 6 LV segments in the radial and longitudinal axis. To measure longitudinal and radial strain/strain rate during early LV filling, the “reverse peak” algorithm of the Vevo Strain software was utilized (de Lucia et al., 2019). We reported these parameters as reverse radial/longitudinal strain/strain rate. LV dyssynchrony was determined in radial and longitudinal using 3 different methods: (a) maximum T2P delay between the earliest and the latest segment, (b) T2P variation, defined as the STD of T2P over all 6 segments, (c) STD of [T2P/RR interval] for each segment (de Lucia et al., 2019). Note, RR interval was obtained with Vevo Strain Software.

HW, BW, and HW/BW were measured at the end of the study for all groups. Statistical analyses for echocardiographic and gravimetric parameters: comparisons between multiple groups were analyzed by linear mixed effect model with group, time and their interactions as fixed effects, and animal as the random effects (random intercept model). We used the lme4 R package for linear mixed effect model and the emmeans R package for the subsequent pair‐wise comparisons between groups at each time points with Tukey's multiple testing correction. Other analysis used one‐way ANOVA with Tukey's multiple comparison tests. A *p*‐value < 0.05 was considered statistically significant. Statistical analyses were performed using Prism 8.4.3 (GraphPad Software Inc.) or R version 4.0.0.

### 4.3 Global metabolomics profiling

#### 4.3.1 Metabolite detection

We performed high‐resolution LC–MS analysis for global (untargeted) metabolite profiling. Forty plasma samples were sent to the University of Washington's Northwest Metabolomics Research Center in Seattle, WA. Specifically, plasma samples were collected from 10 mice from Sham_Young group, 8 mice from Sham_Old group, 11 mice from I/R_Young group, and 11 mice from I/R_Old group. Samples were prepared, acquired, processed, and analyzed as previously described (Ederer et al., 2018; Navarro et al., 2019).

##### Sample preparation

Frozen plasma samples were thawed at 4°C, and proteins were precipitated by mixing 50 µl plasma with 250 µl cold methanol. After 20 min incubation at −20°C, the mixture was centrifuged at 20,800 *g* for 10 min, 4°C. The supernatant was transferred into a clean 2.0 ml Eppendorf tube and dried in an Eppendorf Vacufuge (Brinkmann Instruments, Westbury, NY) at 30°C. The residue was reconstituted in 200 µl H_2_O:acetonitrile (4:6, v/v) and centrifuged at 20,800 *g* for 5 min, 4°C to remove solid residue. The resulting solution (160 µl) was transferred to 2 ml LC vial for MS analysis. Along with the samples, a blank (mobile phase) has been analyzed twice together with a QC (Quality Control) sample for data quality check. QC sample was prepared by pooling 20 µl from each sample.

##### Data acquisition

The data were acquired on an Agilent 1200 SL LC‐6520 Quadrupole‐Time of Flight (Q‐TOF) MS coupled with an Agilent 1260 Infinity Liquid Chromatography. The separation was performed using a Waters XBridge BEH Amide column (15 cm × 2.1 mm, 2.5 µm, serial n^o^ 01273722215406). Mobile phase consisted of (A) H_2_O:acetonitrile (95:5, v/v) with 5 mM ammonium acetate and 0.1% acetic acid, and (B) H_2_O:acetonitrile (5:95, v/v), 5 mM ammonium acetate and 0.1% acetic acid. Gradient elution was performed as follows: 94%–78% of B from 0.0–6.5 min, 78%–39% B from 6.5–12.0 min, at 39% B until 18.5 min, restored to 94% B from 18.5–19.0 min, and maintained 94% B from 19.0–35.0 to equilibrate the LC column for the next injection. The flow rate was set to 0.3 mL min^−1^, column temperature at 35°C and injection volume of 5 µl, followed by H_2_O:acetonitrile (5:95, v/v) needle wash for 10 s. The ESI conditions were as follows: Electrospray ion source ESI Agilent Jet Stream Technology in positive ionization mode; voltage 3.8 kV; desolvation temperature 325°C; cone flow 10 L/h; desolvation gas flow 600 L/h; nebulizer pressure 45 psi, N_2_ was used as drying gas; MS scan rate of 1.03 spectra/s across the range m/z 60–1000. Data were acquired in both positive and negative ionization mode using MassHunter Data Acquisition Workstation v. B.06.01.6157 software (Agilent Technologies).

In order to support the putative annotation from LC‐MS analysis, the samples were also injected on an SCIEX ExionLC AC‐X500 Q‐TOF MS/MS system (AB Sciex, Framingham, MA), using TurbolonSpray ion source in negative and positive ion mode. LC separation was performed using the same stationary phase Waters XBridge BEH Amide column (15 cm × 2.1 mm, 2.5 µm, serial n^o^ 01303902417908), and similar gradient elution condition as described for the data acquisition on the Agilent LC‐MS experiment. Mass measurements were recorded using Data Dependent Acquisition (DDA). The acquisition parameters of the mass spectrometer were: curtain gas of 30, ionspray voltage of 4000 V, ion source gas 1 of 70 psi, ion source gas 2 of 80 psi, MS mass range 60–1000 m/z, maximum candidate ions of 10, intensity threshold 200 counts/s, dynamic background subtraction, isotopic exclusion of ±4 Da, MS/MS mass tolerance of ±10 ppm, MS/MS mass range 20–1000 m/z, collision energy 25 eV with collision spread of 10 eV, and the utilization of exclusion list produced by ions detected in blank injections, with intensity threshold >200 counts. Mass calibration was achieved using the integrated calibrant delivery system (CDS).

##### Data processing

Data processing was performed using Progenesis Qi software v. 2.2.5826.42898 (Nonlinear Dynamics, Newcastle, UK). Peak alignment was carried out taking a QC run as the reference. Peak‐peaking was performed using sensitivity and chromatographic peak width at 3 and 0.05, respectively. The retention time limit was set 2.0–18.0 min. The resulting data were filtered considering +1 charge state in positive ion mode (−1 in negative mode). Possible adduct ions were defined based on previous knowledge on urine LC‐MS untargeted analysis, as follows: [M + H]^+^, [M + Na]^+^, [M + NH_4_]^+^, [2M + H]^+^, [2M + Na]^+^, [2M + NH_4_]^+^, in (+) ESI mode, and [M − H]^−^, [M + CH_3_COO − H]^−^, [M − H_2_O − H]^−^, [M + Cl]^−^, [2M − H]^−^, [M + Na − 2H]^−^, [M + K − 2H]^−^, [2M + CH_3_COO − H]^−^, and [2M + CH_3_COO − H]^−^ in (−) ESI mode. The adduct ions were grouped into mass features through peak deconvolution. Peak annotation was firstly performed by searching metabolites from MS database (Human Metabolome Database) using accurate *m*/*z* measurements from the full‐scan data. We set the *m*/*z* tolerance of 10 ppm, 90% of isotopic similarity, and elemental composition filter of C, H, O, N, P and S. Tandem MS fragmentation pattern was also matched to METLIN MS/MS database for structural assignment. We used only empirical fragments, precursor ion tolerance of 10 ppm, 90% of isotopic similarity, elemental composition filter similar to MS analysis, and a fragment tolerance of 30 ppm.

#### 4.3.2 Data analysis

Analysis of the dataset was performed using R (version 3.6.1). Global LC‐MS provided measures of 603 features with 0.4% missing data from positive mode, and 433 features with 1.8% missing data from negative mode in a total of 40 samples. All the features with ≥5% missingness or with ≥30% coefficient of variation in QC samples were excluded, leaving 585 and 388 features in positive and negative modes, respectively. Positive mode and negative mode data were analyzed separately. We imputed the remaining missing values using the K‐nearest neighbors imputation method implemented in the R impute package (Troyanskaya et al., 2001). The log_2_‐transformed feature abundance was median normalized prior to imputation.

In order to remove batch effects and other unwanted variation in the data, we used the Bioconductor SVA package to identify and estimate surrogate variables (SVs), while keeping the group of interest (Leek & Storey, 2007). We fit a linear model to the normalized and imputed data to detect the group differences in feature abundance using the Bioconductor limma package, while controlling for the estimated SVs as covariates in our model (Ritchie et al., 2015). Similarly, we fit a separated linear model to detect if there was a linear relationship between feature abundance and the cardiac function variables within each group while controlling for the estimated SVs as covariates in our model. The limma package uses empirical Bayes moderated statistics, which improves power by ‘borrowing strength’ between metabolites in order to moderate the residual variance (Smyth, 2004). We used the Benjamini–Hochberg multiple testing method to control the FDR and selected metabolites at an FDR of 10%. For the unsupervised learning, the estimated SVs were removed using the limma “remove‐Batch‐Effect” function prior to the principal component analysis (PCA) and clustering analysis. To identify metabolic pathways whose activity could explain the distribution of *m*/*z* and retention times among the LC‐MS features associated with group differences, we used mummichog version 1.0.10 (Li et al., 2013). Mummichog searched known metabolic pathways for potential enrichment among *m*/*z* and retention time data. In this case, mummichog was provided sets of features, which showed significant effect from the linear model described above. The metabolic model Mouse (Mus musculus, BioCyc 17.0) was used to test the enrichment of pathways and networks. One hundred permutations of the data were performed by mummichog to estimate the null distribution. Instrument accuracy was set at 5 ppm.

## 5 LIMITATIONS

One limitation of our study may lie on the collection of non‐fasting plasma samples. The purpose of our study was to perform an unbiased evaluation of circulating metabolome. Previous studies have shown that HF is a ketosis‐prone state as blood ketone body levels were significantly higher during the fasting (Lommi et al., 1997). Moreover, serum concentrations of the ketone bodies have been shown to change between patients with different type and grades of HF (Zordoky et al., 2015). As we expect (1) this to potentially affect our results as we were studying a particularly susceptible population such as aged mice with heart disease and (2) that some other metabolites in addition to ketone body may be biasedly influenced by fasting in ischemic groups, we decided to collect plasma samples without overnight fasting. Our goal was to understand daily circulating metabolomic profile rather than fasting‐related effects. Hence, we consistently collected plasma samples from all different groups at the same daily time (10 a.m.–12 p.m.). Our mice were housed under a standard 12 h light/12 h dark cycle. Under this cycle, mice have been shown to reliably consume the majority of their food during the dark, with short negligible sessions of feeding during the light (Ellacott et al., 2010). We confirmed this in our all the groups studied (data not shown). We found a clear separation between young and old groups, as previously described and, we do not expect our approach to have been a major issue.

Another limitation of our study is the lack of female mice in our groups. We are aware that gender is an important biological factor in the pathophysiology of heart HF during aging. However, we did not include female mice in our study as we were specifically focused on the evaluation of the effect of age and myocardial ischemia rather than sex on plasma metabolomic profiling. Of note, we decided to study male rather than female mice as HF has a higher prevalence in older man rather older women (Benjamin et al., 2019).

Of note, we believe the reason of not detecting all the putative metabolites in MS/MS analysis may be related to less sensitivity of Data Dependent Acquisition (DDA) in detecting low abundance metabolites than full‐scan MS (Guo & Huan, 2020).

## CONFLICT OF INTEREST

The authors declare no competing financial interests.

## AUTHOR CONTRIBUTIONS

CdL and WJK designed the study, developed the experimental design, and wrote the manuscript. CdL, MP, FCN, and DR performed the experiments. EG performed the sham and I/R surgeries. CdL, MP, and LW performed data analyses. DPromislow and DPratico edited the manuscript. All authors discussed the results and commented on the manuscript.

## Supporting information

Fig S1‐S12

Table S1‐S4

Appendix S1

Appendix S2

Appendix S3

Appendix S4

Appendix S5

Appendix S6

Appendix S7

Appendix S8

Appendix S9

## Data Availability

The authors declare that all supporting data and method descriptions are available within the article or from the corresponding author upon reasonable request.
