# Supplementary material for: Effects of myocardial ischemia/reperfusion injury on plasma metabolomic profile during aging
Source: Aging Cell. 2020 Dec 29;20(1):e13284. doi: 10.1111/acel.13284 (PMC7811846; doi:10.1111/acel.13284)

Suppl. Figure 1:

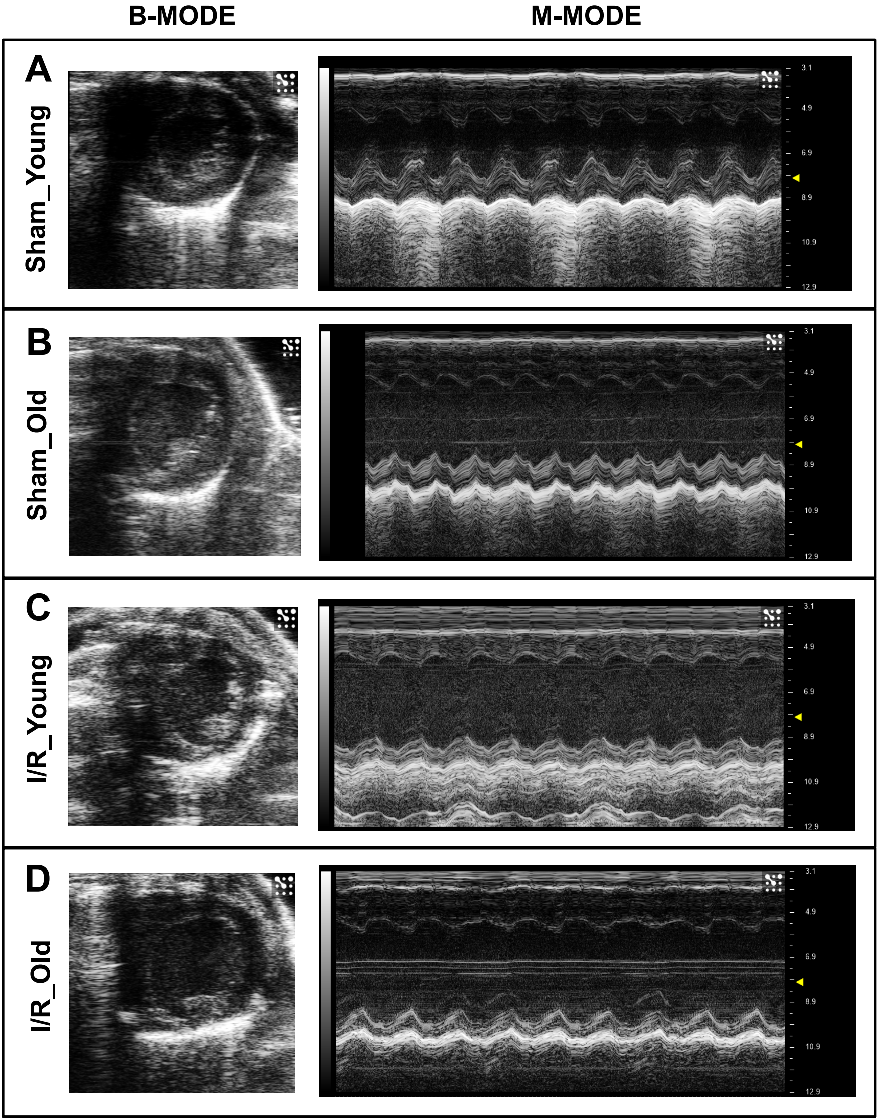

Suppl. Figure 2:

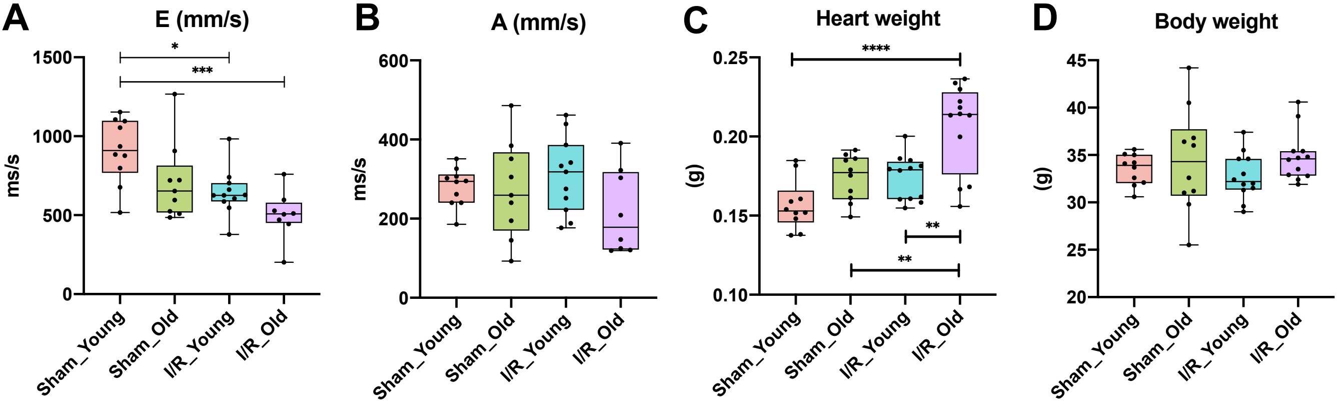

Suppl. Figure 3:

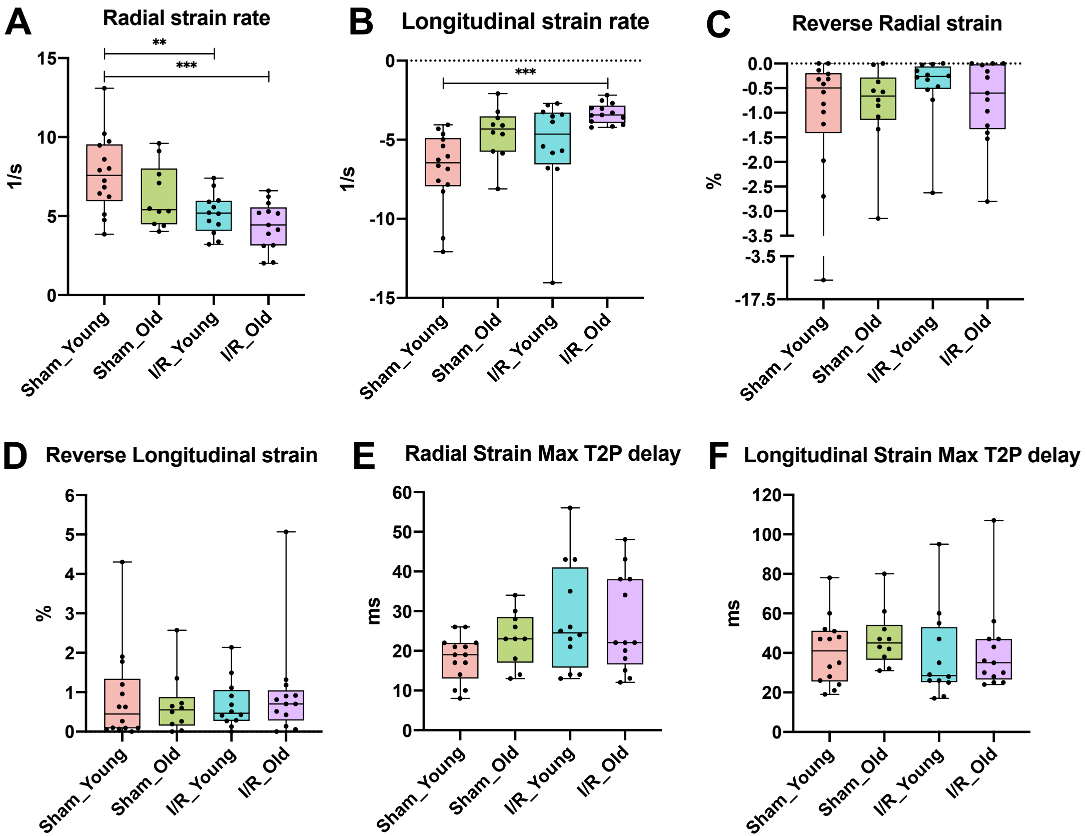

Suppl. Figure 4:

A **Positive mode**

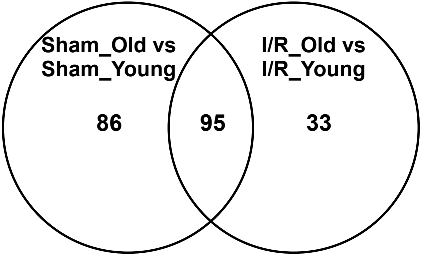

B **Negative mode**

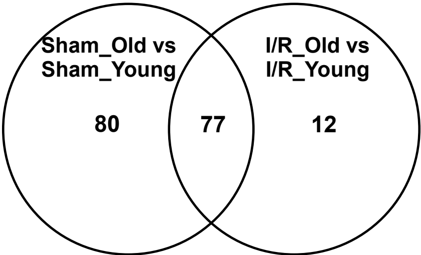

Suppl. Figure 5:

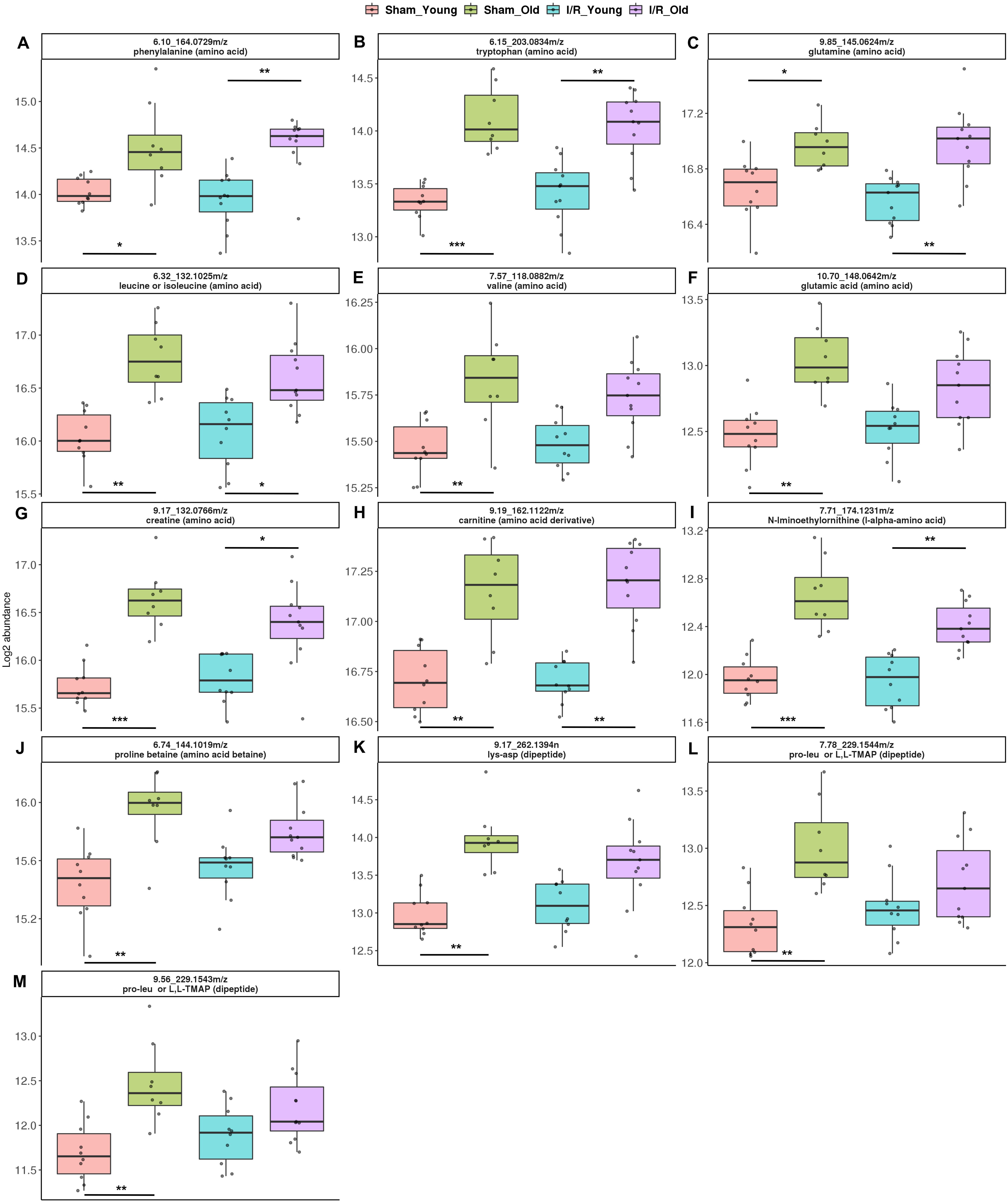

Suppl. Figure 6:

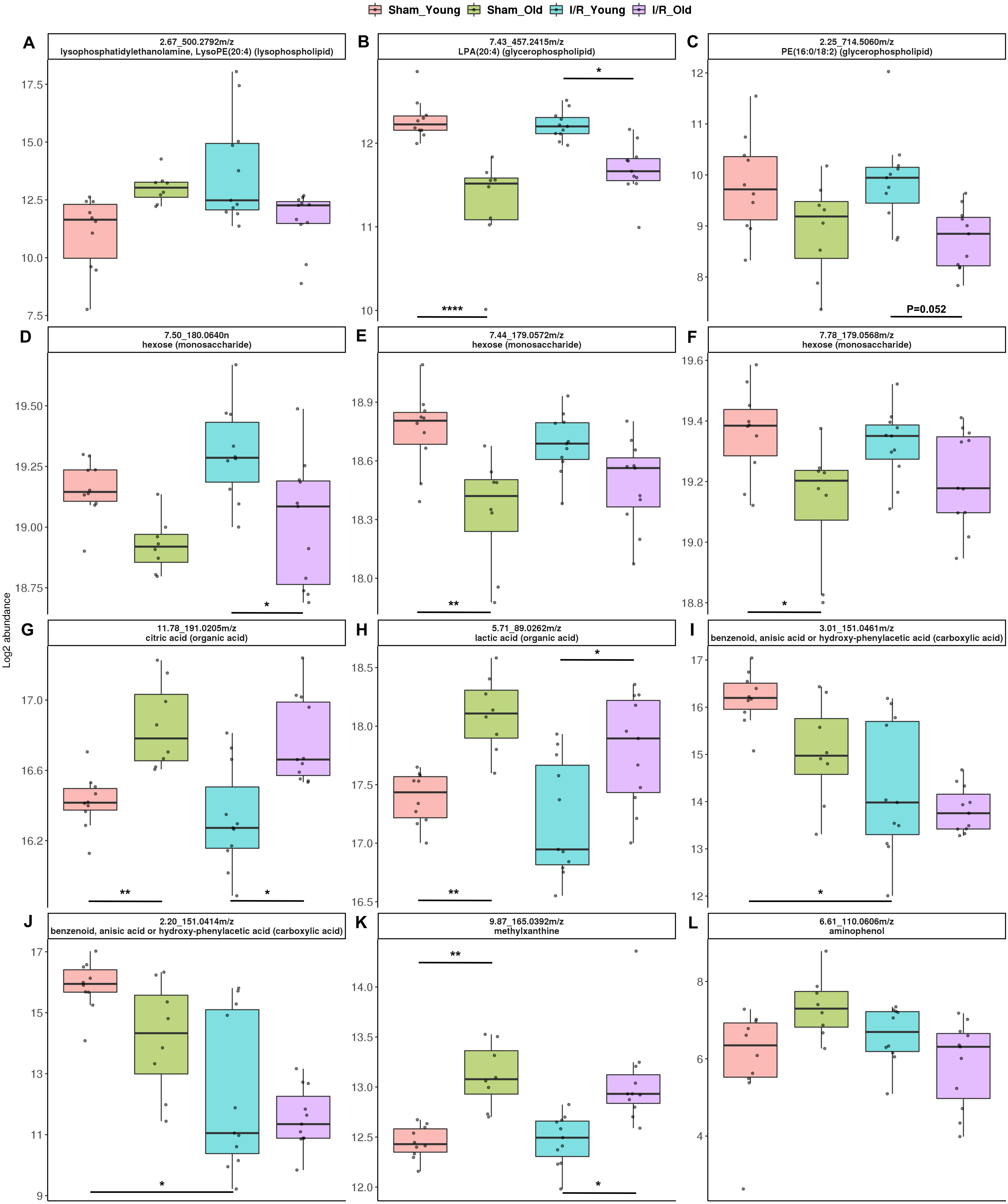

Suppl. Figure 7:

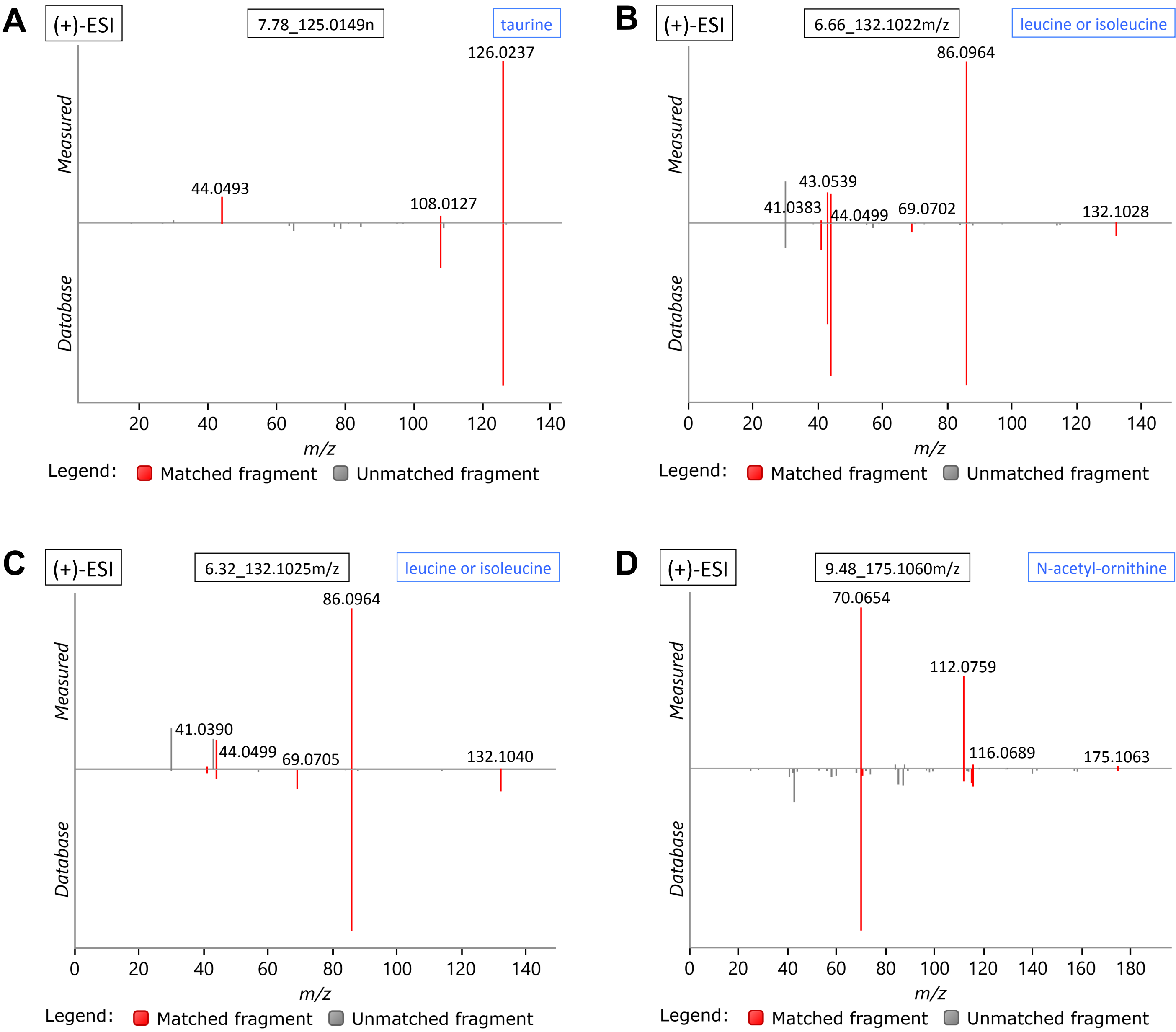

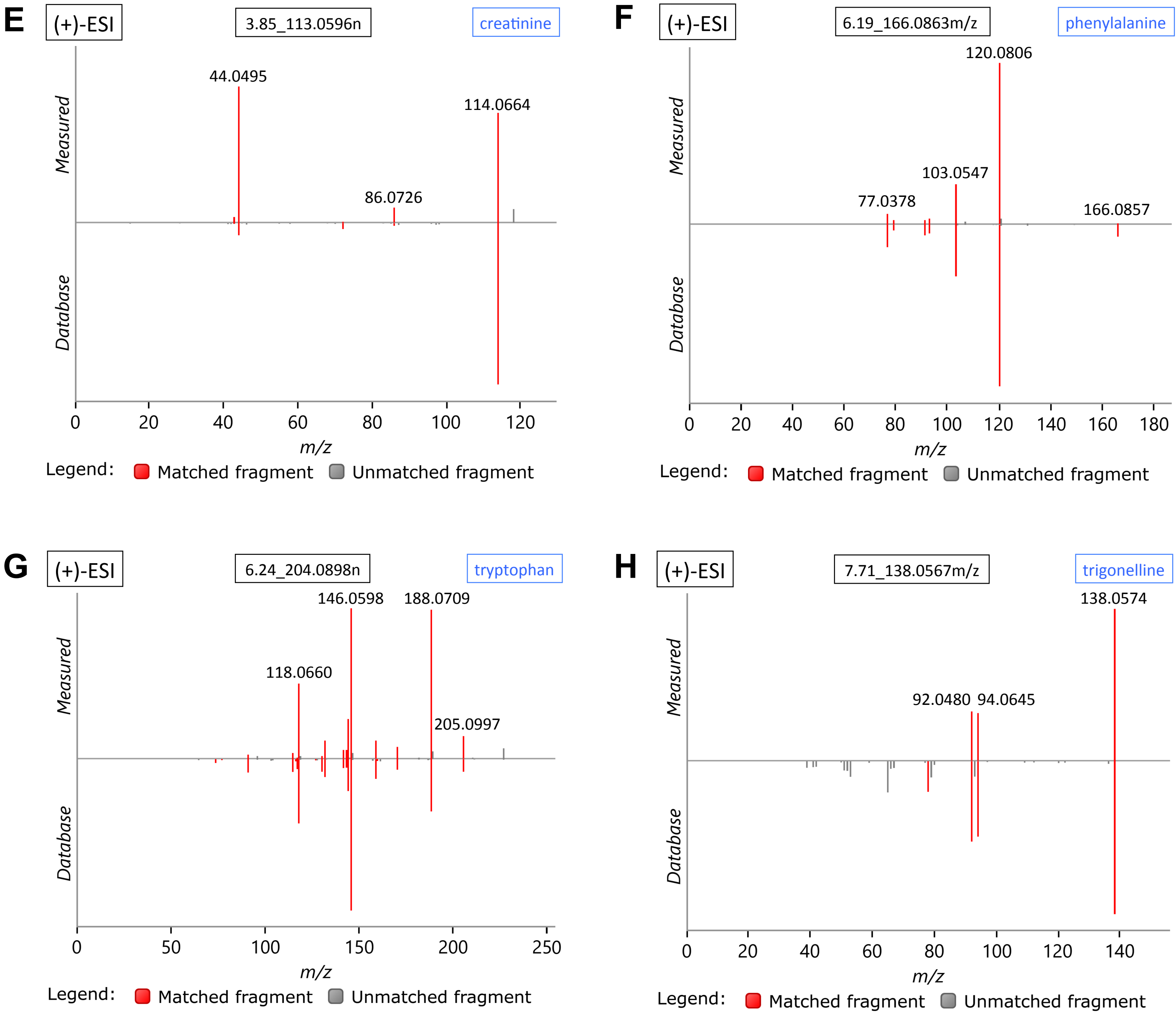
E
(+)-ESI
3.85_113.0596n
creatinine
44.0495
114.0664
86.0726
Measured
Database
0 20 40 60 80 100 120
m/z
Legend: Matched fragment Unmatched fragment
F
(+)-ESI
6.19_166.0863m/z
phenylalanine
120.0806
103.0547
77.0378
166.0857
Measured
Database
0 20 40 60 80 100 120 140 160 180
m/z
Legend: Matched fragment Unmatched fragment
G
(+)-ESI
6.24_204.0898n
tryptophan
146.0598
188.0709
118.0660
205.0997
Measured
Database
0 50 100 150 200 250
m/z
Legend: Matched fragment Unmatched fragment
H
(+)-ESI
7.71_138.0567m/z
trigonelline
138.0574
92.0480
94.0645
Measured
Database
0 20 40 60 80 100 120 140
m/z
Legend: Matched fragment Unmatched fragment

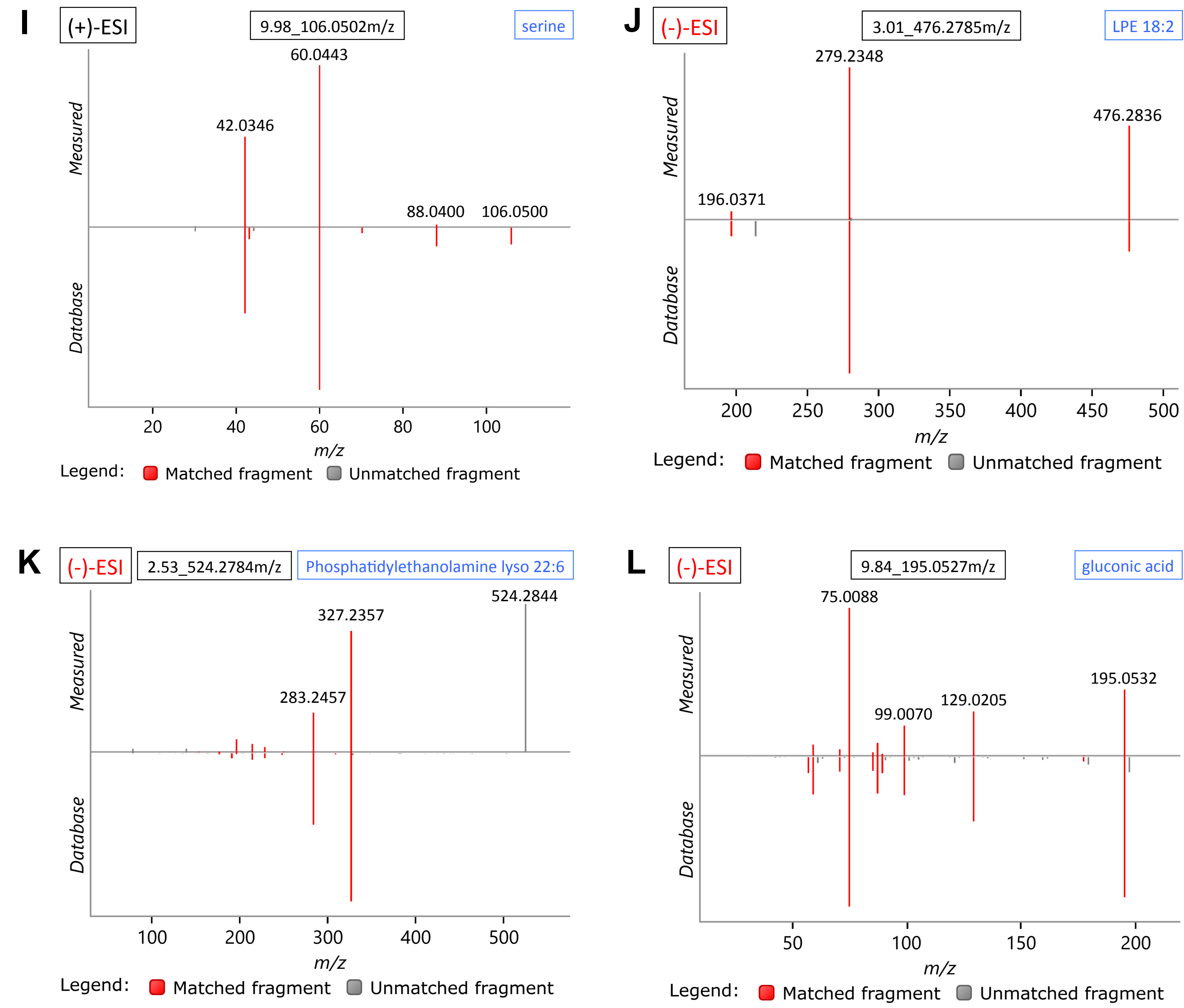

I
(+)-ESI
9.98_106.0502m/z
serine
60.0443
42.0346
88.0400
106.0500
Measured
Database
20
40
60
80
100
m/z
Legend:
Matched fragment
Unmatched fragment
J
(-)-ESI
3.01_476.2785m/z
LPE 18:2
279.2348
476.2836
196.0371
Measured
Database
200
250
300
350
400
450
500
m/z
Legend:
Matched fragment
Unmatched fragment
K
(-)-ESI
2.53_524.2784m/z
Phosphatidylethanolamine lyso 22:6
524.2844
327.2357
283.2457
Measured
Database
100
200
300
400
500
m/z
Legend:
Matched fragment
Unmatched fragment
L
(-)-ESI
9.84_195.0527m/z
gluconic acid
75.0088
195.0532
129.0205
99.0070
Measured
Database
50
100
150
200
m/z
Legend:
Matched fragment
Unmatched fragment

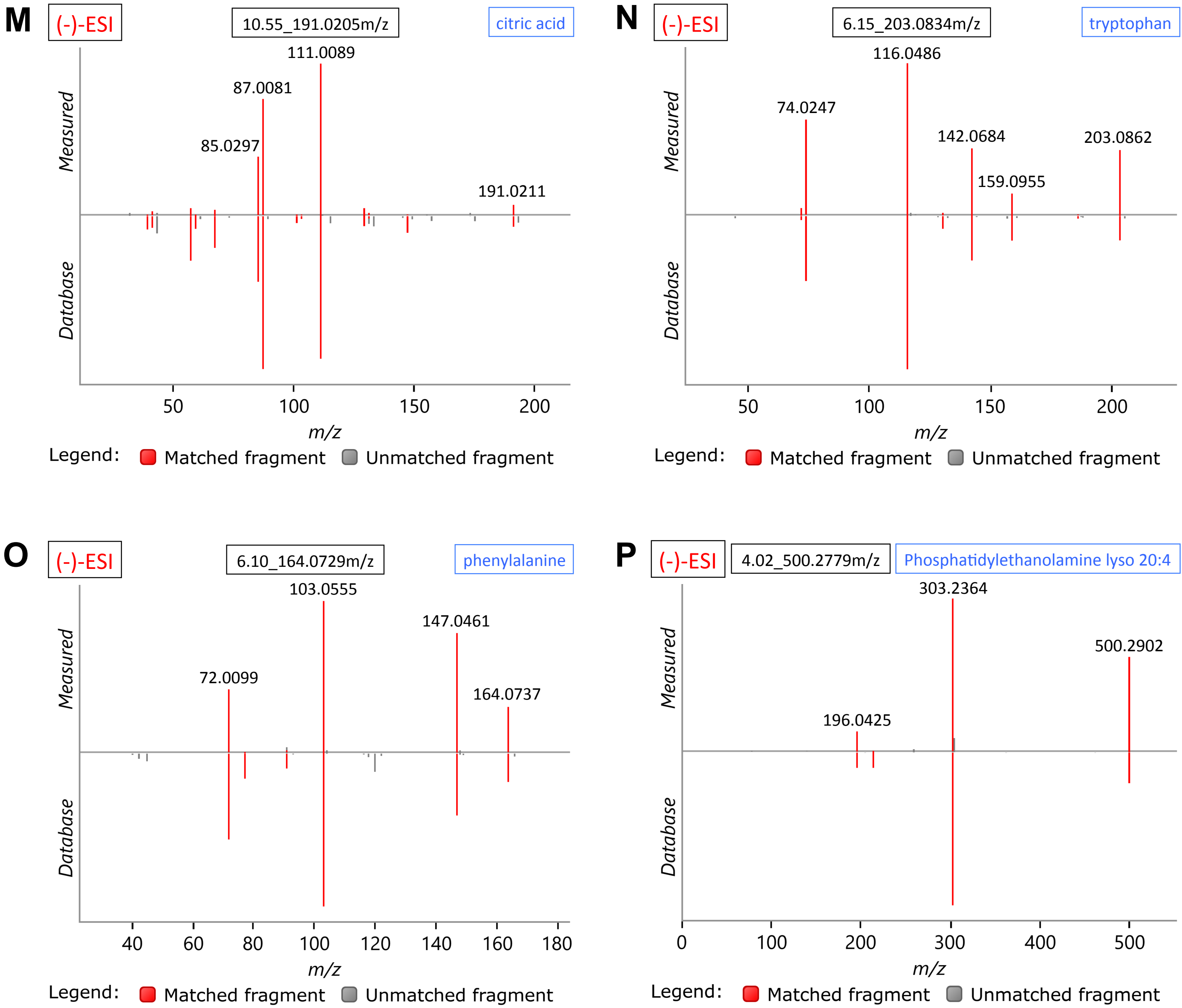
M
(-)-ESI
10.55_191.0205m/z
citric acid
111.0089
87.0081
85.0297
191.0211
Measured
Database
50
100
150
200
m/z
Legend:
Matched fragment
Unmatched fragment
N
(-)-ESI
6.15_203.0834m/z
tryptophan
116.0486
74.0247
142.0684
203.0862
159.0955
Measured
Database
50
100
150
200
m/z
Legend:
Matched fragment
Unmatched fragment
O
(-)-ESI
6.10_164.0729m/z
phenylalanine
103.0555
147.0461
72.0099
164.0737
Measured
Database
40
60
80
100
120
140
160
180
m/z
Legend:
Matched fragment
Unmatched fragment
P
(-)-ESI
4.02_500.2779m/z
Phosphatidylethanolamine lyso 20:4
303.2364
500.2902
196.0425
Measured
Database
0
100
200
300
400
500
m/z
Legend:
Matched fragment
Unmatched fragment

Q

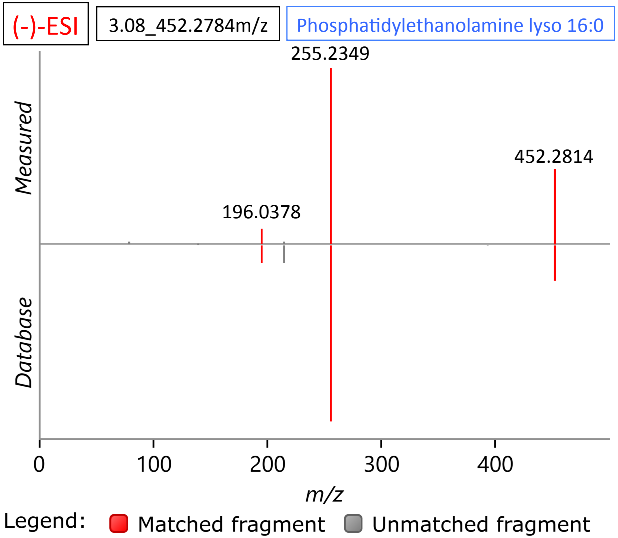

(-)-ESI
3.08_452.2784m/z
Phosphatidylethanolamine lyso 16:0
255.2349
452.2814
196.0378
Measured
Database
0
100
200
300
400
m/z
Legend: Matched fragment Unmatched fragment

R

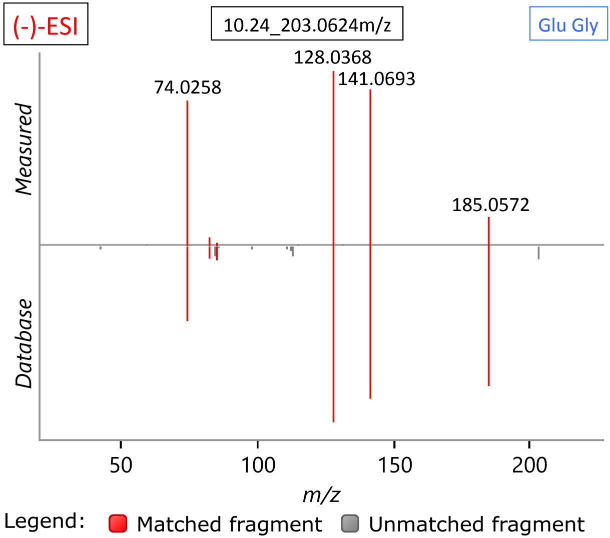

(-)-ESI
10.24_203.0624m/z
Glu Gly
128.0368
141.0693
74.0258
185.0572
Measured
Database
50
100
150
200
m/z
Legend: Matched fragment Unmatched fragment

Suppl. Figure 8:

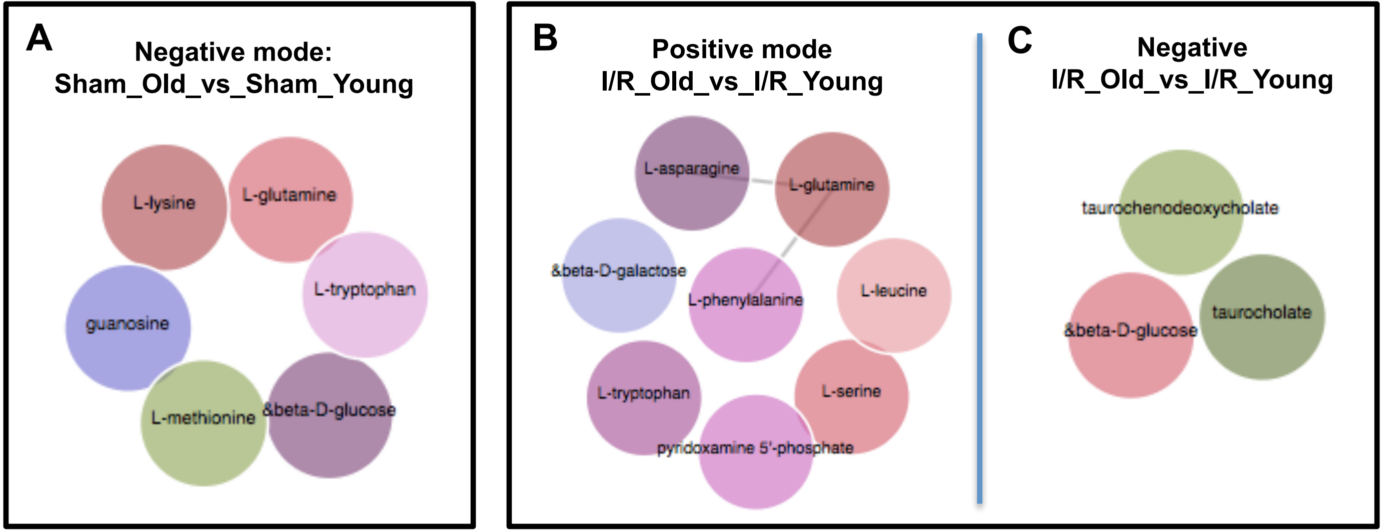

Suppl. Figure 9:

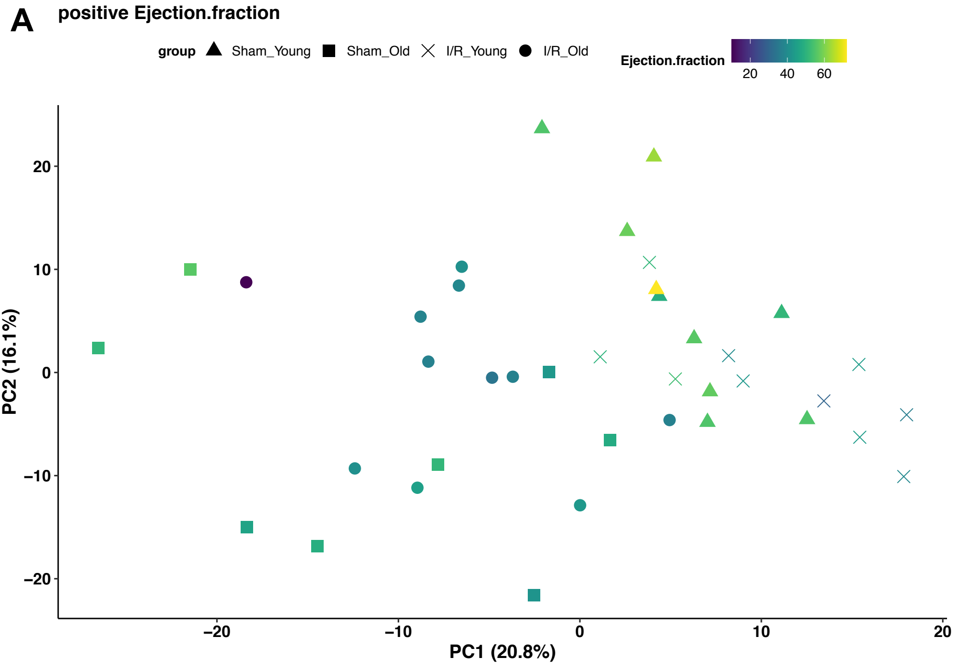

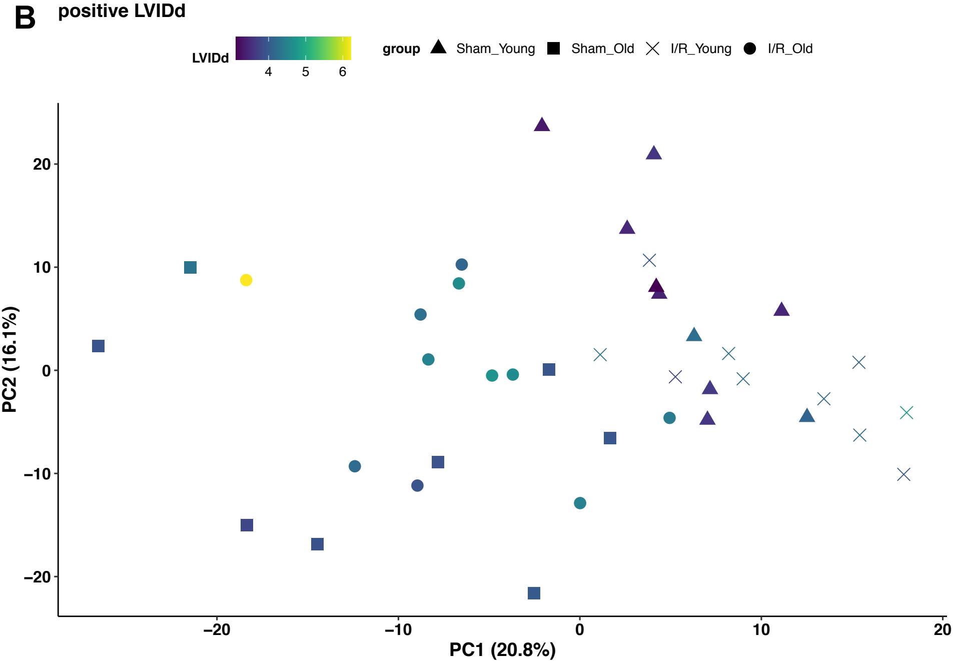

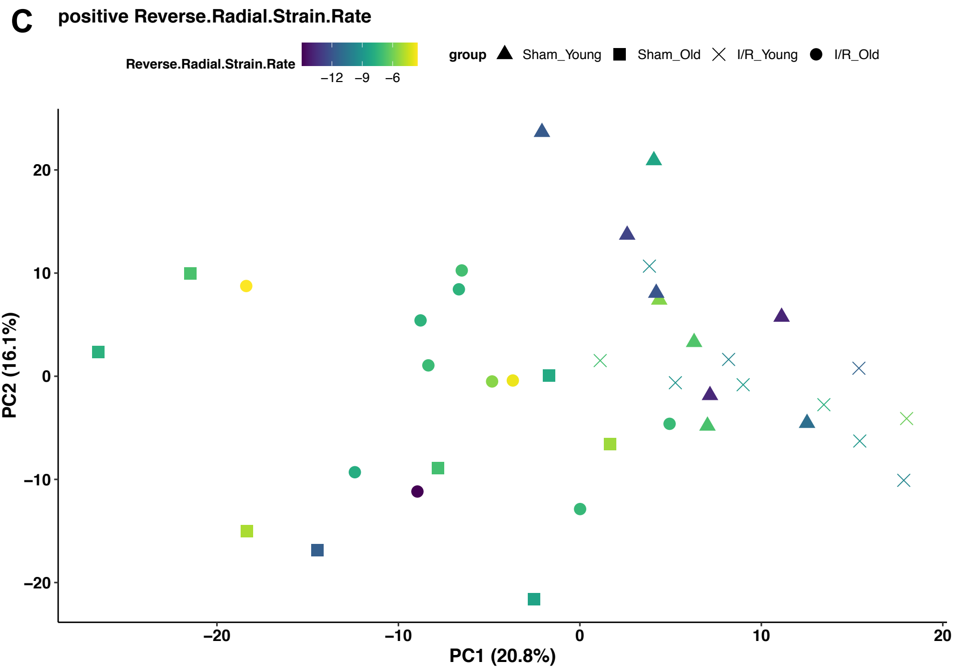
C
positive Reverse.Radial.Strain.Rate
Reverse.Radial.Strain.Rate
−12 −9 −6
group Sham_Young Sham_Old I/R_Young I/R_Old
PC2 (16.1%)
20
10
0
−10
−20
PC1 (20.8%)
−20 −10 0 10 20

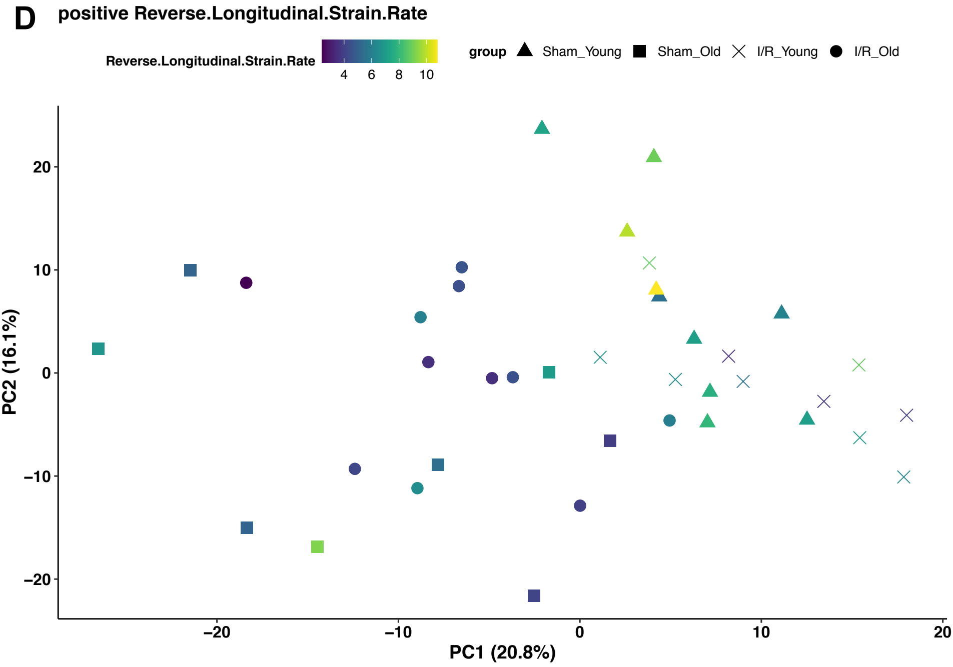
D
positive Reverse.Longitudinal.Strain.Rate
Reverse.Longitudinal.Strain.Rate
4 6 8 10
group Sham_Young Sham_Old I/R_Young I/R_Old
PC2 (16.1%)
20
10
0
−10
−20
PC1 (20.8%)
−20 −10 0 10 20

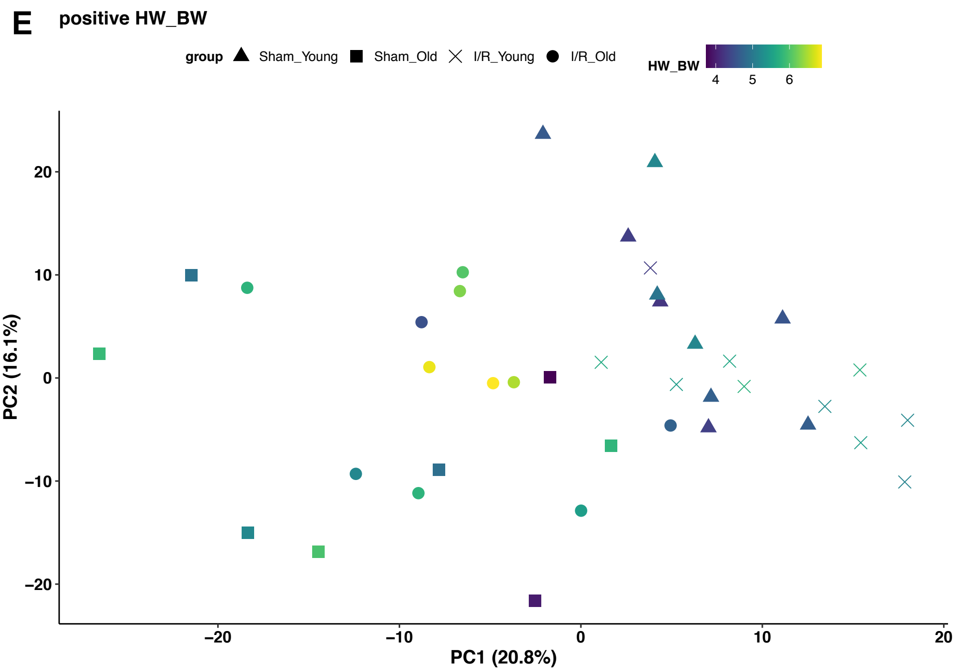

E
positive HW_BW
group
Sham_Young
Sham_Old
I/R_Young
I/R_Old
HW_BW
4
5
6
20
10
0
−10
−20
PC2 (16.1%)
−20
−10
0
10
20
PC1 (20.8%)

Suppl. Figure 10:

A

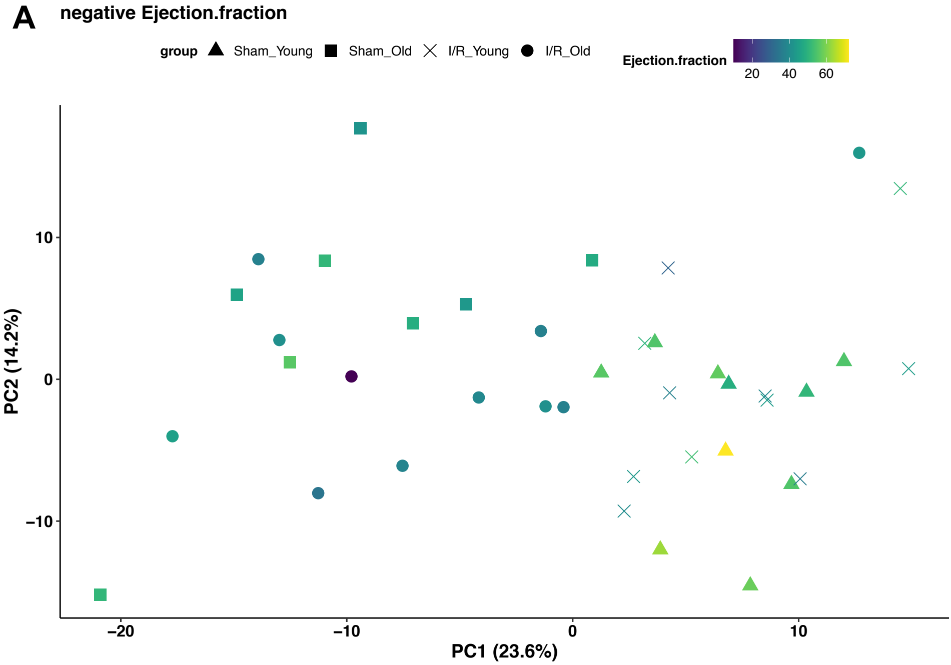

B

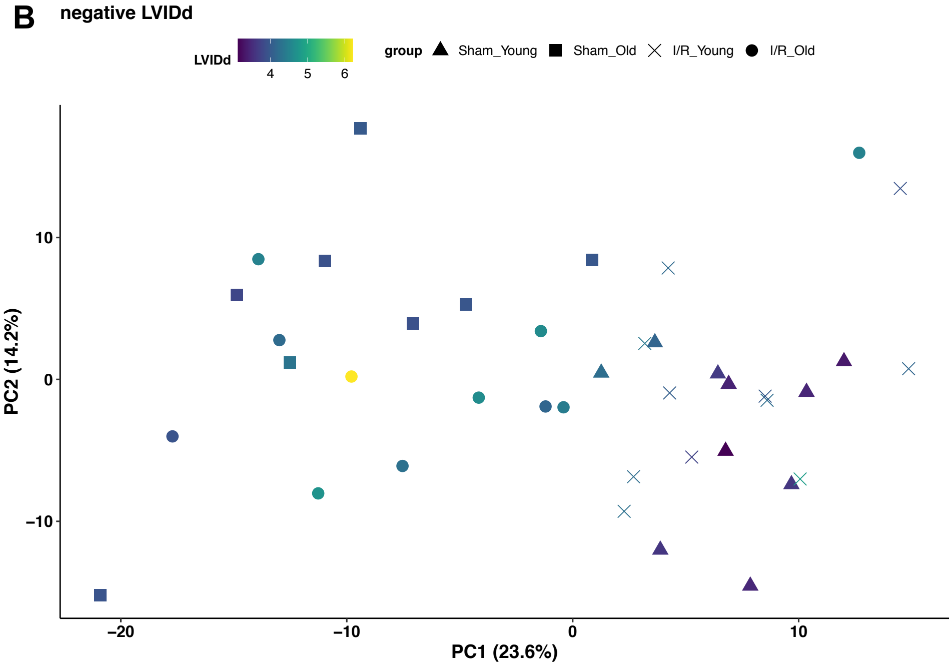

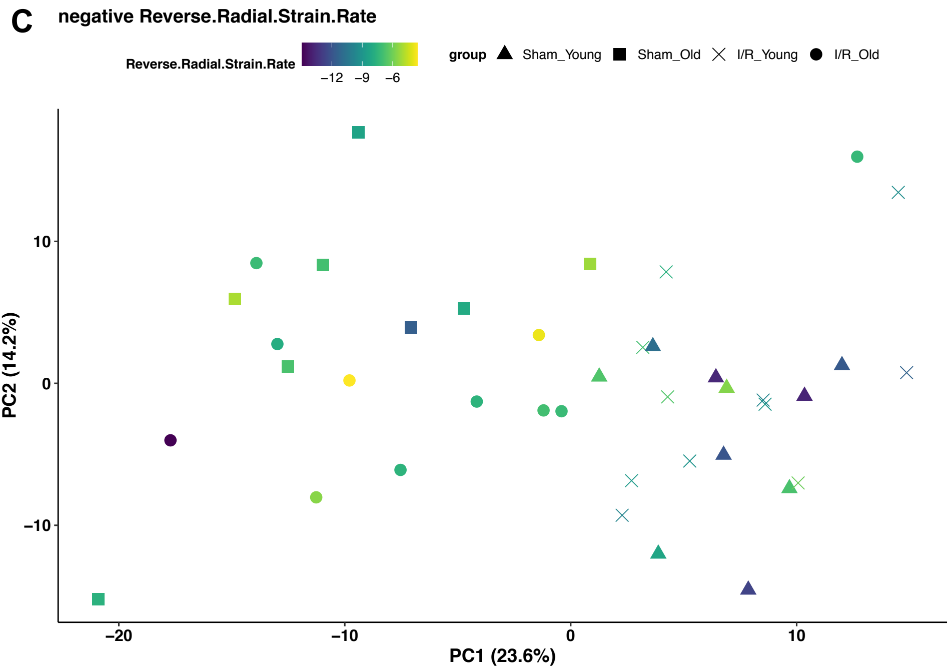
C
negative Reverse.Radial.Strain.Rate
Reverse.Radial.Strain.Rate
−12
−9
−6
group
Sham_Young
Sham_Old
I/R_Young
I/R_Old
PC2 (14.2%)
PC1 (23.6%)
10
0
−10
−20
−10
0
10

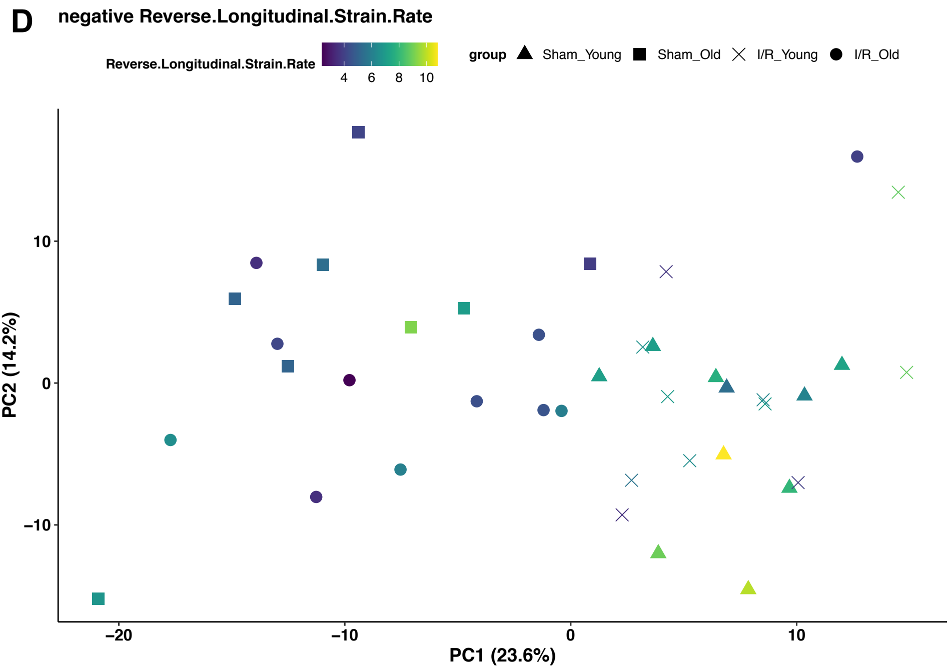
D
negative Reverse.Longitudinal.Strain.Rate
Reverse.Longitudinal.Strain.Rate
4
6
8
10
group
Sham_Young
Sham_Old
I/R_Young
I/R_Old
PC2 (14.2%)
PC1 (23.6%)
10
0
−10
−20
−10
0
10

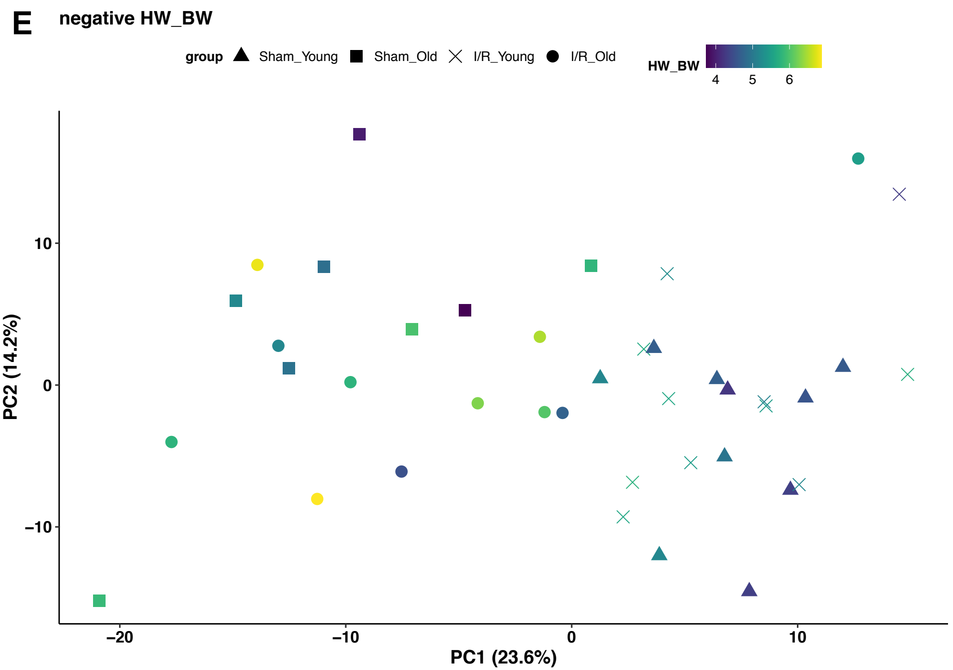

E
negative HW_BW
group
Sham_Young
Sham_Old
I/R_Young
I/R_Old
HW_BW
4
5
6
10
0
−10
−20
−10
0
10
PC2 (14.2%)
PC1 (23.6%)

Suppl. Figure 11:

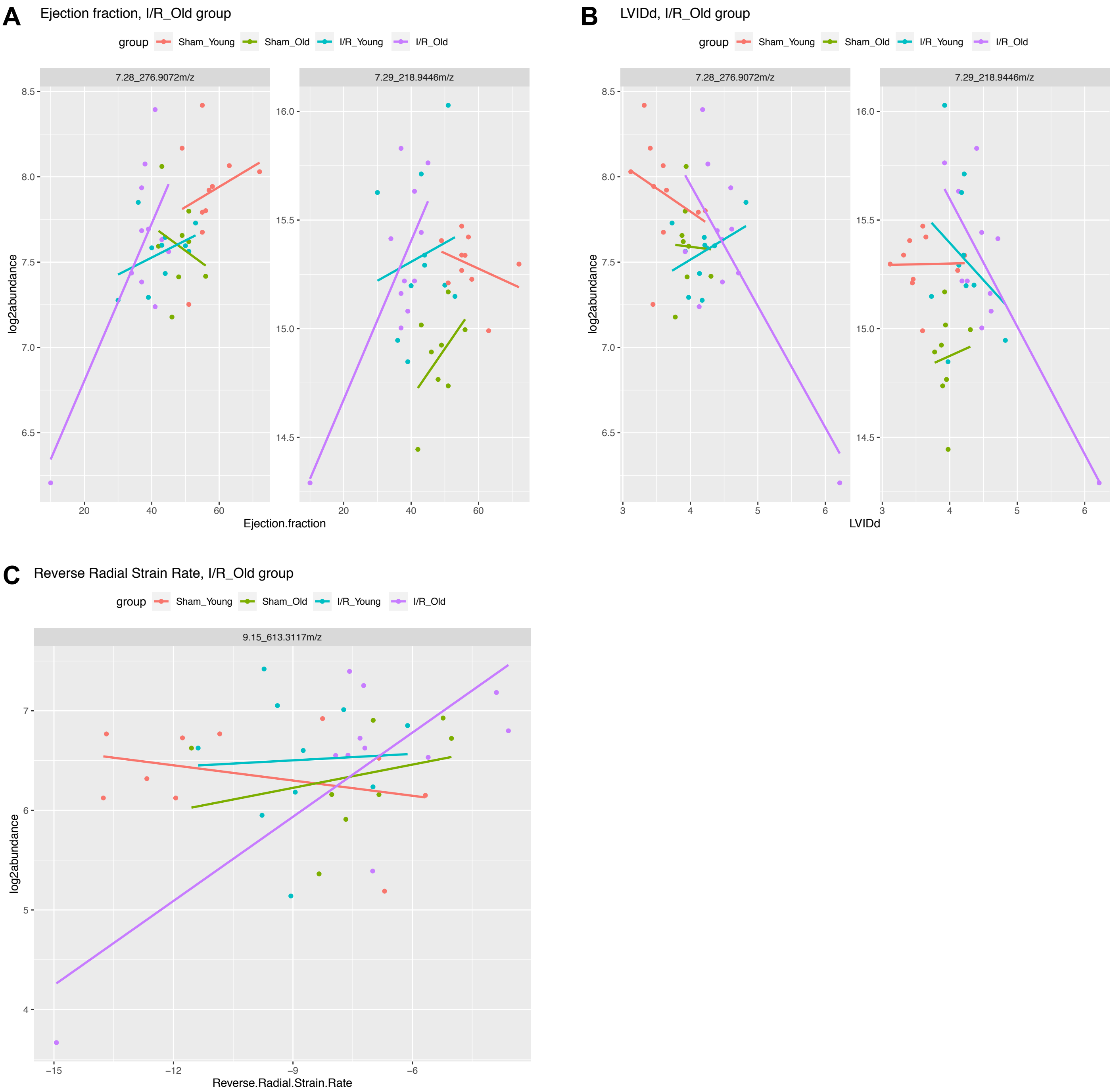

Suppl. Figure 12:

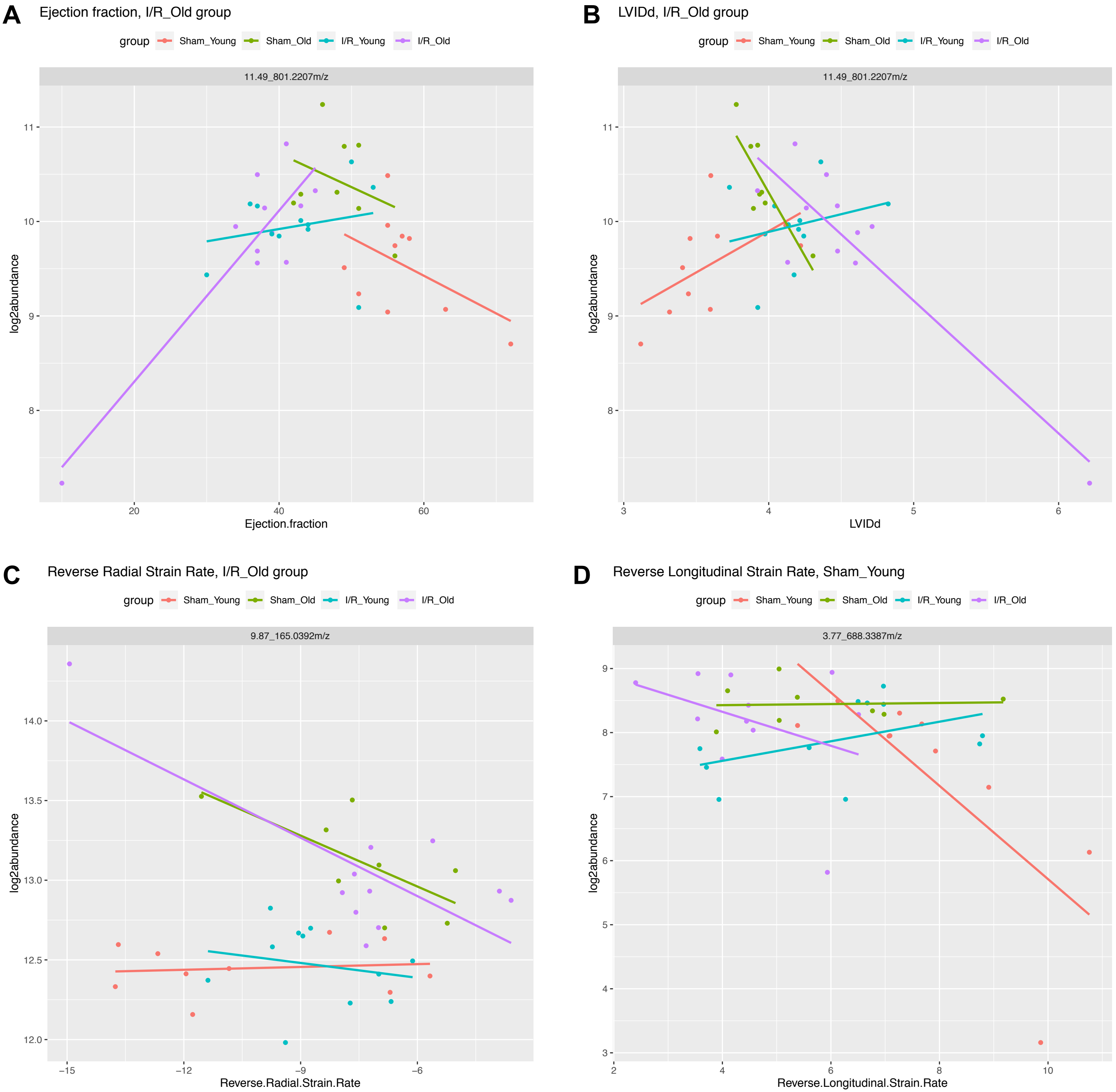

Supplement: Supplementary file 1 — Fig S1‐S12 [file ACEL-20-e13284-s001.pdf]
